# Exploring the Molecular Mechanism of Astragali Radix-Curcumae Rhizoma against Gastric Intraepithelial Neoplasia by Network Pharmacology and Molecular Docking

**DOI:** 10.1155/2021/8578615

**Published:** 2021-10-04

**Authors:** Yuejin Ji, Yajun Liu, Jingyi Hu, Cheng Cheng, Jing Xing, Lei Zhu, Hong Shen

**Affiliations:** Affiliated Hospital of Nanjing University of Chinese Medicine, Nanjing 210029, China

## Abstract

**Background:**

Astragali Radix-Curcumae Rhizoma (ARCR), a classic drug pair, has been widely used for the treatment of gastric intraepithelial neoplasia (GIN) in China. However, the underlying mechanisms of this drug pair are still unknown. Thus, elucidating the molecular mechanism of ARCR for treating GIN is imperative.

**Methods:**

The active components and targets of ARCR were determined from the TCMSP database, and the differentially expressed genes related to GIN were identified from the GSE130823 dataset. The protein-protein interaction (PPI) network and ARCR-active component-target-pathway network were constructed by STRING 11.0 and Cytoscape 3.7.2, respectively. In addition, a receiver operating characteristic curve (ROC) was conducted to verify the key targets, and enrichment analyses were performed using R software. Molecular docking was carried out to test the binding capacity between core active components and key targets.

**Results:**

31 active components were obtained from ARCR, among which 22 were hit by the 51 targets associated with GIN. Gene Ontology (GO) functional enrichment analysis showed that biological process (BP), molecular function (MF), and cellular component (CC) were most significantly enriched in response to a drug, catecholamine binding, and apical part of the cell, respectively. Kyoto Encyclopedia of Genes and Genomes (KEGG) pathway analysis indicated ARCR against GIN through regulation of neuroactive ligand-receptor interaction, nitrogen metabolism, calcium signaling pathway, chemical carcinogenesis-receptor activation, drug metabolism, gap junction, and cancers. In the PPI network, 15 potential targets were identified, of which nine key targets were proven to have higher diagnostic values in ROC. Molecular docking revealed a good binding affinity of active components (quercetin, bisdemethoxycurcumin, and kaempferol) with the corresponding targets (CYP3A4, CYP1A1, HMOX1, DRD2, DPP4, ADRA2A, ADRA2C, NR1I2, and LGALS4).

**Conclusion:**

This study revealed the active components and molecular mechanism by which ARCR treatment is effective against GIN through regulating multipathway, such as neuroactive ligand-receptor interaction, nitrogen metabolism, and calcium signaling pathway.

## 1. Introduction

Gastric cancer (GC) is one of the most common cancers worldwide, ranking fifth for incidence and fourth for mortality globally [1]. Eastern Asia, including China, has the highest incidence rate [2]. In China, the number of new cases of GC was 679,000, and the number of deaths was 498,000 in 2015 [3]. Therefore, preventing the occurrence of GC in China is an arduous and great task.

Based on Lauren classification [4], GC is divided into two major histological types, diffuse and intestinal gastric cancer (IGC) types. Correa et al. proposed a prevailing model for the stages of IGC, wherein normal gastric mucosa progresses to chronic superficial gastritis and then to chronic atrophic gastritis (CAG), intestinal metaplasia, dysplasia, and IGC [5]. As the most serious precancerous lesion of GC, the treatment of gastric intraepithelial neoplasia (GIN) is indispensable for preventing GC. At present, the main therapies for high-grade intraepithelial neoplasia include endoscopic mucosal resection and/or endoscopic submucosal dissection. However, low-grade intraepithelial neoplasia and high-grade intraepithelial neoplasia patients unwilling to undergo endoscopic surgery still lack effective therapies.

Astragali Radix (ARX) and Curcumae Rhizoma (CR) are two kinds of Chinese herbal medicines, which are often chosen to treat tumor-related diseases in the form of a drug pair [6, 7]. Astragali Radix is called Huangqi in Chinese, a representative medicine for tonifying Qi and strengthening the spleen. Curcumae Rhizoma is called Ezhu in Chinese, good at activating blood and resolving stasis. The combination of ARX and CR is mainly responsible for tonifying Qi and activating blood (Yiqi Huoxue), one of the most commonly used treatment methods for GIN in traditional Chinese medicine. Qian et al. found that Yiqi Huoxue Decoction may play a preventive role in gastric precancerous lesions by downregulating the expression of Sonic Hedgehog signaling pathway [8]. In a clinical study, Yiqi Huoxue Fang combined with quadruple therapy can improve the main clinical symptoms, enhance the secretion of gastric mucosa, improve the content of pepsinogen in serum, and reduce the level of inflammatory factors in *Helicobacter pylori* positive CAG patients [9]. Our previous study confirmed that Yiqi Huoxue Fang based on Astragali Radix-Curcumae Rhizoma (ARCR) has a good clinical effect on gastric precancerous lesions [10].

**Figure 1:**
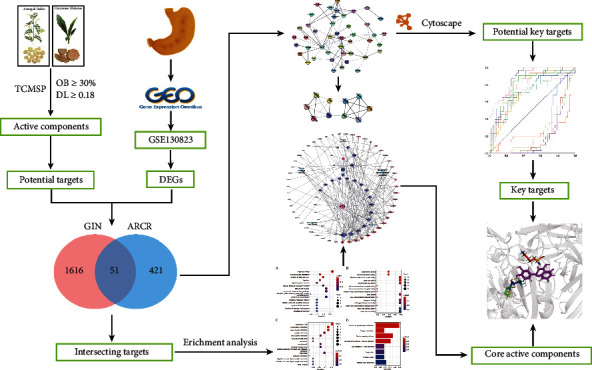
Flowchart of our study.

In order to elucidate the mechanism of ARCR in the treatment of GIN, we explored the key targets and pathways of ARCR against GIN through network pharmacology and molecular docking. The flowchart of our analysis is shown in Figure 1.

## 2. Materials and Methods

### 2.1. Screening the Active Components and Targets for ARCR

The active components were extracted from the Traditional Chinese Medicine Database and Analysis Platform (TCMSP) (https://tcmsp-e.com/) [11]. Oral bioavailability (OB) ≥ 30% and drug-likeness (DL) ≥ 0.18 were set as the thresholds to screen the active components of ARCR in the TCMSP database and then obtain the corresponding targets for each active component. Meanwhile, other active components not retrieved were supplemented by related literature reviews. If the targets of active components cannot be obtained from the TCMSP database, the prediction of the relevant targets was conducted in SwissTargetPrediction [12]. The 2D structures of the active components were downloaded from PubChem (https://pubchem.ncbi.nlm.nih.gov/) [13]. All targets of ARCR were standardized in the UniPort database [14].

### 2.2. Collection of GIN-Related Differentially Expressed Genes (DEGs)

A microarray expression profiling dataset GSE130823 was selected for exploring the DEGs in Gene Expression Omnibus (GEO) [15]. GSE130823, based on the GPL17077 platform, included 98 gastric tissue biopsy samples (47 chronic gastritis, 31 GIN, and 16 IGC). The function of normizeBetweenArray in Limma package [16] was used to normalize GSE130823, and the DEGs were identified using the Limma package in R software. Targets with the cutoff criteria of |log2 FoldChange| > 1 and adjusted *P* value < 0.05 were regarded as DEGs [17].

### 2.3. Construction of Protein-Protein Interaction (PPI) Network and Identification of the Key Targets

The intersecting targets between ARCR and GIN were obtained by ggVennDiagram package in R software, and Veen map was drawn. Then, we imported the intersecting targets into the STRING 11.0 [18], with the species limited to “Homo sapiens” and the minimum required interaction score set as 0.900 and the disconnected nodes hid in the network. Subsequently, the PPI network was visualized and analyzed in Cytoscape 3.7.2 [19], to obtain the core functional modules by Cytoscape MCODE [20] and identify the potential key targets by CytoHubba App [21]. Receiver operating characteristic curve (ROC) analysis was performed, using IBM SPSS version 26, to verify the diagnostic values of key targets in GIN. The expression levels of the key targets were obtained from the GSE130823 dataset. The area under ROC curve (AUC) was calculated, and the statistical standard of AUC ≥ 0.7 and *P* value < 0.05 were used.

### 2.4. Enrichment Analysis of the Intersecting Targets

We use clusterProfiler package [22] to conduct Kyoto Encyclopedia of Genes and Genomes (KEGG) pathway analysis and Gene Ontology (GO) functional enrichment analysis in R software. A bar chart or a bubble diagram was constructed after screening out the first 15 functional categories of biological process (BP), cellular component (CC), molecular function (MF), and KEGG pathway enrichment analysis, respectively. The threshold of adjusted *P* value < 0.05 was considered as the screening criteria.

### 2.5. Network Construction of ARCR-Active Component-Intersecting Target-KEGG Pathway

ARCR, active components, intersecting targets, and signaling pathways were introduced into Cytoscape 3.7.2 software to construct and analyze the network, in order to screen the core active components of ARCR in treating GIN.

### 2.6. Molecular Docking Analysis

Molecular docking analysis of the core active components of ARCR and the key targets was conducted. The molecular structures of the core active components were searched in ZINC database [23], and the 3D structures of the key targets were obtained in PDB database [24]. Use PYMOL to remove water molecules and ligands of the targets. Upload the PDB format files of PYMOL-processed targets and the mol2 format files of the core active components to the SwissDock platform [25] for molecular docking. Use UCSF Chimera and PYMOL to analyze and visualize the results of molecular docking.

## 3. Results

### 3.1. Active Components and Its Corresponding Targets of ARCR

Initially, obtain 89 active components of ARX and 82 active components of CR from the TCMSP database. With the criteria of OB ≥ 30% and DL ≥ 0.18, combining the related literature reviews [26–29], a total of 31 active components were identified, of which 24 were from ARX and eight were from CR (one active component was shared by ARX and CR). A total of 1101 targets were associated with the 31 active components identified in ARCR, of which 822 were associated with ARX and 279 with CR. After removing the overlapping targets, 472 targets were remaining, including 367 from ARX and 212 from CR. The active components of ARCR are shown in Table 1.

### 3.2. DEGs for GIN

The gastric biopsy tissues of 31 GIN patients in the GSE130823 were used as the research object, and 47 chronic gastritis tissues were used as the control group. According to the cutoff criteria of |log2 FoldChange| > 1 and adjusted *P* value < 0.05, a total of 1667 DEGs in the GSE130823 dataset were found to be dysregulated in GIN tissues by the Limma package, including 737 upregulated genes and 930 downregulated genes (Supplementary Table 1).

### 3.3. PPI Network Construction and Key Targets Identification

**Figure 2:**
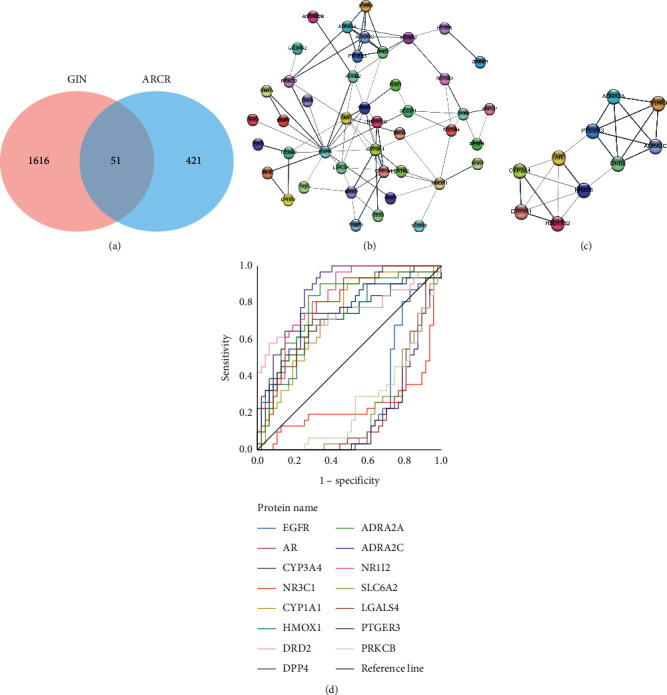
(a) Venn diagram between the targets of ARCR and the DEGs of GIN. GIN: gastric intraepithelial neoplasia; ARCR: Astragali Radix-Curcumae Rhizoma. (b) Protein-protein interaction network of the intersecting targets. Each bubble node represents a protein. The lines among inner nodes display the relationship between different proteins, and the width of lines was based on the strength of data support. (c) A core function module from the PPI network. (d) The results of ROC curve.

Using the R software to draw the Venn map between the targets of ARCR and the DEGs of GIN (Figure 2(a)), we obtained 51 intersecting targets. The STRING 11.0 was used to construct the PPI network of the intersecting targets of ARCR as related to the treatment of GIN. After hiding the isolated nodes, the PPI network contained 44 nodes and 87 edges (Figure 2(b)). One core function module was identified by Cytoscape MCODE (Figure 2(c)), and target proteins with the top 15 degree values were considered as the potential key targets in the PPI network (Table 2). The results of ROC analysis indicated that nine key targets (CYP3A4, CYP1A1, HMOX1, DRD2, DPP4, ADRA2A, ADRA2C, NR1I2, and LGALS4) had higher diagnostic values (Figure 2(d), Table 2).

### 3.4. GO Functional Enrichment Analysis and KEGG Pathway Analysis of Intersecting Targets

**Figure 3:**
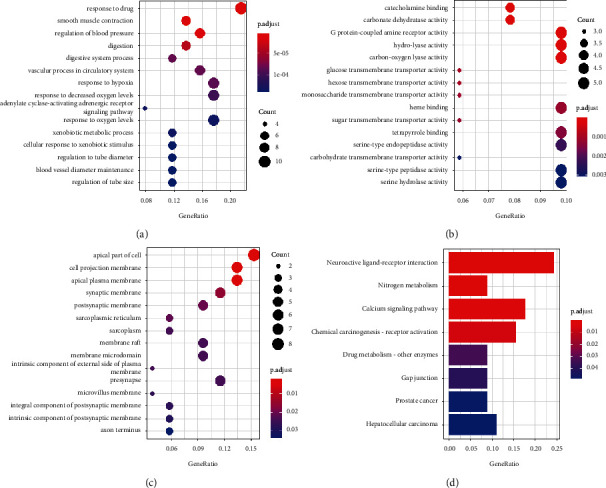
Results of GO enrichment analysis and KEGG pathway analysis of the intersecting targets. (a) Biological process; (b) molecular function; (c) cellular component; (d) Kyoto Encyclopedia of Genes and Genomes pathway analysis.

To further elucidate the biological functions of the 51 intersecting targets, GO enrichment analysis was performed in R software. GO functional enrichment analysis of BP obtained a total of 363 enrichment items, the targets were markedly enriched for response to drug, smooth muscle contraction, regulation of blood pressure, digestion, and digestive system process (Figure 3(a)). MF analysis obtained a total of 64 enrichment items, the targets were significantly involved in catecholamine binding, carbonate dehydratase activity, G protein-coupled amine receptor activity, hydro-lyase activity, and carbon-oxygen lyase activity (Figure 3(b)). CC analysis obtained a total of 25 enrichment items; the targets were mainly enriched in the apical part of the cell, cell projection membrane, apical plasma membrane, synaptic membrane, and postsynaptic membrane (Figure 3(c)). KEGG pathway analysis returned 8 items, including neuroactive ligand-receptor interaction, nitrogen metabolism, calcium signaling pathway, chemical carcinogenesis-receptor activation, drug metabolism, gap junction, and cancers (Figure 3(d)).

### 3.5. ARCR-Active Component-Intersecting Target-KEGG Pathway Network

**Figure 4:**
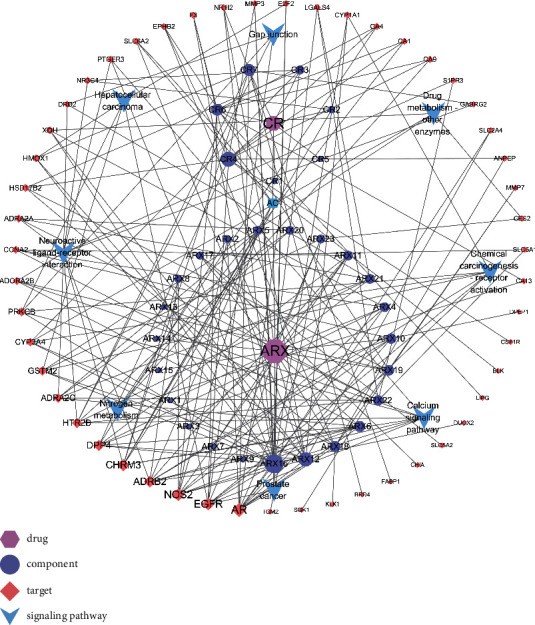
Network of ARCR-active component-intersecting target-signaling pathway. The size of the graph represents the degree value. The larger the graph, the higher the degree value. ARX16: quercetin; ARX12: kaempferol; CR4: bisdemethoxycurcumin.

Cytoscape 3.7.2 was used to construct the network of ARCR-active component-intersecting target-KEGG pathway, involving 92 nodes and 213 edges (Figure 4). Analysis of the topology of the network showed that ARX16 (quercetin) has the highest degree value (degree = 18, betweenness centrality = 0.1795, and closeness centrality = 0.4396) and indicated that quercetin is the most pivotal ingredient among the active components of ARCR in treating GIN, followed by CR4 (bisdemethoxycurcumin, degree = 13, betweenness centrality = 0.1249, closeness centrality = 0.3625), and ARX12 (kaempferol, degree = 12, betweenness centrality = 0.0704, closeness centrality = 0.4081). In the network, nine active components (CR1, CR5, ARX1, ARX3, ARX7, ARX9, ARX13, ARX14, and ARX15) without intersecting targets information were deleted. Thus, a total of 22 active components of ARCR played roles in treating GIN, of which 17 were from ARX and 6 from CR (one active component was shared by ARX and CR).

### 3.6. Molecular Docking of Core Active Components and Key Targets

**Figure 5:**
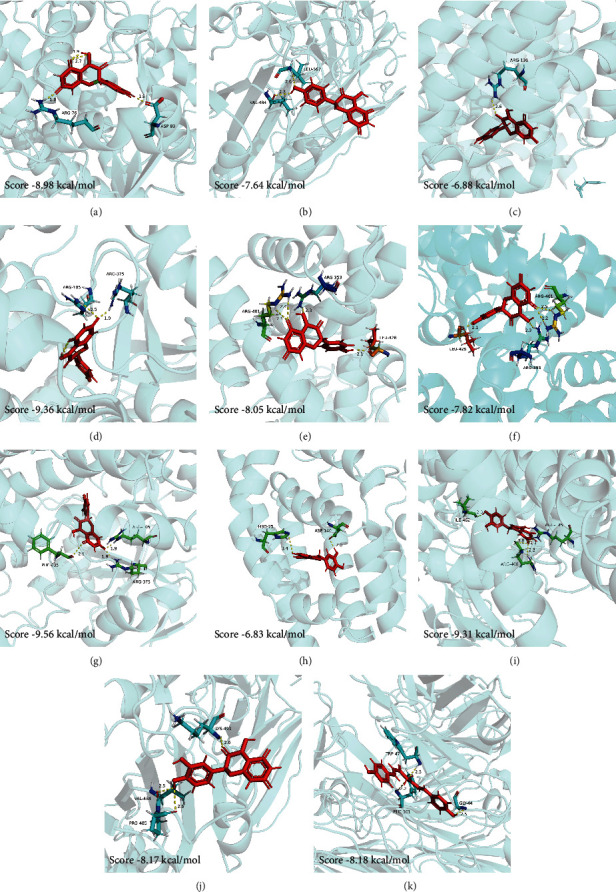
Molecular docking diagrams for three core active components with their corresponding key targets. (a) Kaempferol-CYP1A1; (b) kaempferol-DPP4; (c) kaempferol-HMOX1; (d) kaempferol-CYP3A4; (e) kaempferol-NR1I2; (f) quercetin-NR1I2; (g) quercetin-CYP3A4; (h) quercetin-HMOX1; (i) quercetin-CYP1A1; (j) quercetin-DPP4; and (k) bisdemethoxycurcumin-DPP4.

Three core active components (quercetin, bisdemethoxycurcumin, and kaempferol) and each of the corresponding key targets (CYP3A4, CYP1A1, HMOX1, DRD2, DPP4, ADRA2A, ADRA2C, NR1I2, and LGALS4) were used as ligands and receptors, respectively. When the binding energy is less than 5 kcal/mol, the ligand is regarded to bind well to the receptor [30]. The molecular docking diagrams and binding energy results are shown in Figure 5.

As shown in Figure 5, all active components can bind well to their corresponding key targets, indicating quercetin, bisdemethoxycurcumin, and kaempferol are the core active components of ARCR in treating GIN, and the therapeutic effect might be mediated by nine key targets (CYP3A4, CYP1A1, HMOX1, DRD2, DPP4, ADRA2A, ADRA2C, NR1I2, and LGALS4).

## 4. Discussion

GIN is the most serious gastric precancerous lesion, and the incidence rate of GC in GIN patients is significantly higher than that in CAG and IM patients [31]. Therefore, the treatment of GIN is an effective measure to prevent GC. Previous basic studies mainly focused on different types of solid tumors (gastric cancer). The continuous process from inflammation to GIN is often ignored; however, GIN is closely related to the occurrence and development of GC. Thus, we should pay more attention to the transformation process of inflammation to GIN.

In the study, a total of 22 active components of ARCR were screened, among which three core active components (quercetin, bisdemethoxycurcumin, and kaempferol) were identified. Key pathways were mainly involved in neuroactive ligand-receptor interaction, nitrogen metabolism, calcium signaling pathway, drug metabolism, gap junction, and cancer. Moreover, in PPI network and ROC analysis, nine targets (CYP3A4, CYP1A1, HMOX1, DRD2, DPP4, ADRA2A, ADRA2C, NR1I2, and LGALS4) were selected as the key targets that may play important roles in ARCR against GIN.

Quercetin is one of the main active components of Astragali Radix, belonging to a kind of flavonoid and widely found in various Chinese herbal medicines. It has anti-inflammatory effects by inhibiting the secretion of inflammatory cytokines and chemokines [32, 33]. A population-based study in Sweden showed that high dietary quercetin intake is inversely related to the risk of noncardia gastric adenocarcinoma and the protection appears to be particularly strong for women exposed to oxidative stress, such as tobacco smoking [34], indicating quercetin may have an important preventive effect on GC. Previous studies had further confirmed the protective effect of quercetin on gastric mucosal epithelial cells in in vivo and in vitro experiments [35–37]. As a demethoxy derivative of curcumin, bisdemethoxycurcumin is derived from Curcumae Rhizoma and has a wide range of biological characteristics. Luo et al. [38] established a human gastric adenocarcinoma xenograft model in vivo using nude mice. After intervention with bisdemethoxycurcumin, specific indicators of mitochondrial dysfunction were detected in the mitochondria: the reduction in adenosine triphosphate generation, the inner mitochondrial membrane potential, and augmentation of reactive oxygen species production and cytochrome c, indicating that bisdemethoxycurcumin can reduce the growth of gastric adenocarcinoma by inducing mitochondrial dysfunction. Kaempferol is a natural flavonoid that is widely existing in many fruits, vegetables, and Chinese herbal medicines [39]. Several studies have shown that a certain concentration of kaempferol can inhibit the cell cycle of a variety of tumor cells, induce tumor cell apoptosis, and thereby inhibit tumor cell/tissue invasion and metastasis [40]. A subsequent study confirmed kaempferol can activate the IRE1-JNK-CHOP signaling from cytosol to nucleus, and G9a inhibition can promote autophagic cell death in GC cells, producing anticancer effects [41]. Previous studies confirmed that the three core active components have antigastric cancer properties; however, there is still a lack of relevant research on gastric precancerous lesions, which should be further improved.

After ROC diagnostic analysis, CYP3A4 and CYP1A1 were considered as the two key targets with the highest degree values, speculating that they may play a crucial role in ARCR against GIN. CYP3A4 and CYP1A1 are members of the cytochrome P450 superfamily of enzymes. A recent study found that the expression of CYP3A4 might be related to the potential carcinogenic transformation of CAG to GC, which may be biomarkers to predict the progression of CAG and the poor prognosis of GC [42]. As an isoenzyme of the CYP family I, CYP1A1 mainly participates in the oxidative conversion of the xenobiotics. The importance of CYP1A1 derives from its preferential extrahepatic activities and widespread substrate specificity mainly of xenobiotics [43]. However, its metabolic reactions may accidentally lead to producing the highly reactive compounds that form DNA adducts, contributing to mutagenesis and carcinogenesis [44, 45]. The neuroactive ligand-receptor interaction pathway is the most significantly enriched signaling pathway, involving three key targets (DRD2, ADRA2A, and ADRA2C). G protein-coupled receptors (GPCRs) are large and diverse signaling receptors that are altered in various cancers and regulate cancer progression. Dopamine receptors, a class of GPCRs, have 5 subtypes named DRD1–DRD5 [46]. In general, dopamine receptors are involved in neurological disorders; however, studies indicated that dopamine receptors are also involved in tumor progression [47–49]. Previous studies suggested that dopamine receptor D2 (DRD2) expression can be used to evaluate the prognosis of patients with gastric or esophageal cancer [49, 50]. Moreover, targeting DRD2 has been a novel therapeutic strategy in tumor-related diseases [51–53]. Thus, DRD2 may play an important role in GC. However, no previous studies provided experimental evidence of how DRD2 functions in GIN. ADRA2A and ADRA2C are the alpha-2-adrenergic receptors, a type of adrenergic receptors, and also are the paralogs of DRD2. Studies have confirmed that the expression of ADRA2A is related to the risk of breast cancer [54, 55]. Therefore, these key targets may become candidate genes for the treatment of GIN.

The study still has several limitations. First, the results of the study are not verified in experiments. However, the study is the first to explore the mechanism of ARCR in the treatment of GIN via network pharmacology and molecular docking and will provide theoretical references for subsequent experiments in proven models. Second, the quality of the microarray expression profiling dataset could not be evaluated. However, the ROC diagnostic analysis was used to verify the key targets, which improved the stability and feasibility of the results.

## 5. Conclusions

In summary, the study used network pharmacology and molecular docking to systematically elaborate the mechanism of ARCR in the treatment of GIN. The results indicated that quercetin, bisdemethoxycurcumin, and kaempferol are the core active components, which may generate therapeutic effects on GIN through targets named CYP3A4, CYP1A1, HMOX1, DRD2, DPP4, ADRA2A, ADRA2C, NR1I2, and LGALS4. The study revealed the material basis of ARCR and the possible mechanism for the treatment of GIN and unearthed the potential targets of ARCR in the treatment of GIN, which will provide a reference for follow-up experimental research.

## Figures and Tables

**Table 1:** Active components of ARCR.

Number	Mol ID	Molecule name	OB%	DL
CR1	MOL000915	(1S,10S), (4S,5S)-germacrone-1(10), 4-diepoxide	30.48	0.18
CR2	MOL000902	Curcumol	103.55	0.13
CR3	MOL000906	Wenjine	47.93	0.47
CR4	MOL000940	Bisdemethoxycurcumin	77.38	0.26
CR5	—	Germacrone	-	-
CR6	MOL000090	Curcumin	5.15	0.41
CR7	—	Curdione	-	-
ARX1	MOL000211	Mairin	55.38	0.78
ARX2	MOL000239	Jaranol	50.83	0.29
ARX3	MOL000033	Cholest-5-en-3alpha-ol	36.23	0.78
ARX4	MOL000354	Isorhamnetin	49.60	0.31
ARX5	MOL000371	3,9-di-O-Methylnissolin	53.74	0.48
ARX6	MOL000378	7-O-Methylisomucronulatol	74.69	0.30
ARX7	MOL000379	9,10-Dimethoxypterocarpan-3-O-*β*-D-glucoside	36.74	0.92
ARX8	MOL000380	Methylnissolin	64.26	0.42
ARX9	MOL000387	Bifendate	31.10	0.67
ARX10	MOL000392	Formononetin	69.67	0.21
ARX11	MOL000417	Calycosin	47.75	0.24
ARX12	MOL000422	Kaempferol	41.88	0.24
ARX13	MOL000433	FA	68.96	0.71
ARX14	MOL000439	Isomucronulatol-7,2′-di-O-glucoside	49.28	0.62
ARX15	MOL000442	1,7-Dihydroxy-3,9-dimethoxy pterocarpene	39.05	0.48
ARX16	MOL000098	Quercetin	46.43	0.28
ARX17	MOL000374	5′-Hydroxyiso-muronulatol-2′,5′-di-O-glucoside	41.72	0.69
ARX18	MOL000398	Isoflavanone	109.99	0.30
ARX19	MOL000438	(3R)-3-(2-Hydroxy-3,4-dimethoxyphenyl) chroman-7-ol	67.67	0.26
ARX20	MOL000407	Astragaloside IV	22.5	0.15
ARX21	MOL000401	Astragaloside I	46.79	0.11
ARX22	MOL000403	Astragaloside II	46.06	0.13
ARX23	—	2-(Chloromethyl)-4-(4-nitrophenyl)-1,3-thiazole	-	-
AC	MOL000296	Hederagenin	36.91	0.75

CR1–CR7 represent the active components of CR; ARX1–ARX23 represent the active components of ARX; and AC is the active components shared by ARX and CR. OB: oral bioavailability; DL: drug-likeness.

**Table 2:** Topology attributes of 15 potential key targets and AUC of the ROC analysis.

Gene name	Protein name	Degree value	Betweenness centrality	Closeness centrality	AUC
EGFR	Epidermal growth factor receptor	15	0.4020073	0.5308642	0.254
AR	Androgen receptor	10	0.16157789	0.48314607	0.202
CYP3A4	Cytochrome P450 3A4	9	0.14518632	0.4673913	0.710
NR3C1	Glucocorticoid receptor	9	0.12596012	0.48314607	0.245
CYP1A1	Cytochrome P450 1A1	8	0.08766745	0.43	0.723
HMOX1	Heme oxygenase 1	7	0.11612398	0.37719298	0.733
DRD2	Dopamine receptor D2	6	0.04369106	0.38392857	0.755
DPP4	Dipeptidyl peptidase 4	6	0.11595851	0.38392857	0.859
ADRA2A	Alpha-2A adrenergic receptor	6	0.01645573	0.3495935	0.787
ADRA2C	Alpha-2C adrenergic receptor	6	0.01645573	0.3495935	0.754
NR1I2	Nuclear receptor subfamily 1 group I member 2	6	0.02101245	0.3495935	0.823
SLC6A2	Sodium-dependent noradrenaline transporter	6	0.13161388	0.36752137	0.215
LGALS4	Galectin-4	5	0.02235789	0.41346154	0.771
PTGER3	Prostaglandin E2 receptor EP3 subtype	5	0.03093548	0.37068966	0.185
PRKCB	Protein kinase C beta type	5	0.09021468	0.41346154	0.256

AUC: the area under the ROC curve.

## Data Availability

The data used in the study are available from the corresponding author upon request.

## Abbreviations

ARCR: Astragali Radix-Curcumae Rhizoma
GIN: Gastric intraepithelial neoplasia
PPI: Protein-protein interaction
ROC: Receiver operating characteristic curve
GO: Gene Ontology
BP: Biological process
MF: Molecular function
CC: Cellular component
KEGG: Kyoto Encyclopedia of Genes and Genomes
IGC: Intestinal gastric cancer
CAG: Chronic atrophic gastritis
ARX: Astragali Radix
CR: Curcumae Rhizoma
TCMSP: Traditional Chinese Medicine Database and Analysis Platform
OB: Oral bioavailability
DL: Drug-likeness
GEO: Gene Expression Omnibus
AUC: The area under the ROC curve.

## Supplementary Materials

Supplementary Materials Supplementary Table 1: Differentially expressed genes of GIN in GSE130823.
