# Supplementary material for: Exploring the Molecular Mechanism of Astragali Radix-Curcumae Rhizoma against Gastric Intraepithelial Neoplasia by Network Pharmacology and Molecular Docking
Source: Evid Based Complement Alternat Med. 2021 Oct 4;2021:8578615. doi: 10.1155/2021/8578615 (PMC8505068; doi:10.1155/2021/8578615)
Supplement: Supplementary Materials — Supplementary Table 1: Differentially expressed genes of GIN in GSE130823. [file 8578615.f1.pdf]

TABLE 1: The differentially expressed genes of GIN in GSE130823

Protein name	log2 FoldChange	P value	adjusted P value	change
CHP2	5.902967	2.92E-08	4.29E-06	up
OLFM4	5.227284	3.90E-06	0.000123	up
HOXB13	4.991963	1.57E-10	2.88E-07	up
CPS1	4.806236	2.20E-07	1.62E-05	up
ADH4	4.46519	3.49E-07	2.19E-05	up
CDX1	4.462033	8.46E-09	2.09E-06	up
CLDN3	4.423281	9.04E-10	8.06E-07	up
REG4	4.282192	9.50E-08	9.18E-06	up
ANXA13	4.254453	2.75E-07	1.87E-05	up
HOXA13	4.139407	1.24E-08	2.57E-06	up
CLDN2	4.134212	2.50E-09	1.17E-06	up
SERPINB5	4.132474	2.07E-10	2.88E-07	up
CDH17	4.020024	1.70E-07	1.37E-05	up
KLK8	3.959541	3.55E-09	1.37E-06	up
CLRN3	3.950696	9.43E-09	2.25E-06	up
KLK6	3.935545	2.19E-09	1.17E-06	up
SLC39A5	3.879958	6.00E-08	6.91E-06	up
TRIM54	3.848517	6.86E-10	7.09E-07	up
LEFTY1	3.830799	8.74E-07	4.17E-05	up
HOXA11-AS1	3.759289	3.00E-09	1.26E-06	up
XLOC_006390	3.688811	1.15E-08	2.44E-06	up
KLK7	3.674303	3.31E-08	4.74E-06	up
CEACAM6	3.628526	4.68E-08	5.94E-06	up
CXorf61	3.574797	3.44E-07	2.18E-05	up
GPA33	3.55202	2.19E-07	1.62E-05	up
CDX2	3.55095	5.68E-08	6.72E-06	up
HOXA10	3.516088	1.39E-08	2.78E-06	up
ONECUT2	3.500239	5.01E-09	1.60E-06	up
GUCY2C	3.437024	2.29E-08	3.64E-06	up
OTC	3.42232	2.76E-07	1.87E-05	up
FLJ22763	3.416648	1.59E-05	0.000348	up
SPINK4	3.408739	3.16E-05	0.000581	up
XLOC_006025	3.401275	1.79E-09	1.13E-06	up
MYO7B	3.400391	4.98E-08	6.27E-06	up
BTNL3	3.315126	0.000589	0.005415	up
GRIN2D	3.311716	7.87E-09	2.02E-06	up
MSLN	3.305785	2.12E-07	1.61E-05	up
XLOC_006277	3.295837	2.11E-08	3.52E-06	up
MLN	3.293822	6.89E-05	0.00105	up
CLCA1	3.253583	0.000119	0.001603	up
PPP1R1B	3.240868	7.23E-11	2.29E-07	up

ANPEP	3.237321	0.000187	0.002236	up
TRIM29	3.233292	1.51E-08	2.88E-06	up
VIL1	3.21181	5.95E-07	3.19E-05	up
HOXA11	3.208146	8.00E-08	8.22E-06	up
LOC389332	3.184201	3.02E-09	1.26E-06	up
SI	3.160716	0.000197	0.002323	up
DMBT1	3.137306	2.35E-05	0.000463	up
KRT17	3.123753	1.92E-07	1.50E-05	up
HOXB9	3.11173	1.13E-06	5.01E-05	up
HOTTIP	3.109337	1.06E-08	2.34E-06	up
LOC157860	3.078004	8.10E-10	7.88E-07	up
NOS2	3.056294	1.68E-06	6.73E-05	up
MEP1A	2.966576	0.00015	0.001903	up
CNDP1	2.956509	4.34E-07	2.58E-05	up
XLOC_009934	2.949801	1.73E-05	0.00037	up
PDZK1	2.903163	1.54E-07	1.28E-05	up
GPR128	2.89862	3.90E-05	0.000677	up
MUC17	2.890403	2.69E-07	1.85E-05	up
ZG16	2.876243	0.000767	0.006669	up
TM4SF20	2.869049	4.34E-06	0.000133	up
HOXA9	2.816252	1.51E-07	1.27E-05	up
DEFA6	2.805074	0.001064	0.008616	up
FGFBP1	2.766943	1.95E-06	7.40E-05	up
CDH3	2.744336	3.07E-07	2.01E-05	up
C20orf118	2.742095	9.44E-07	4.40E-05	up
TM4SF4	2.724351	3.97E-06	0.000124	up
F12	2.714777	1.03E-09	8.23E-07	up
SLC3A1	2.700479	4.47E-06	0.000135	up
LRRC19	2.692685	2.28E-05	0.000452	up
LY6D	2.692654	0.000805	0.006936	up
UCA1	2.69124	2.76E-06	9.43E-05	up
LOC100507511	2.679005	2.51E-09	1.17E-06	up
TFF3	2.678081	6.73E-09	1.89E-06	up
SLC17A4	2.668691	6.63E-06	0.00018	up
DPP4	2.652453	1.64E-08	2.99E-06	up
CAMK2N1	2.637984	1.20E-09	8.96E-07	up
RARRES1	2.621305	3.79E-07	2.32E-05	up
CLDN15	2.614874	3.46E-07	2.18E-05	up
CHRNA5	2.604248	4.05E-09	1.45E-06	up
ADORA2B	2.580009	1.91E-06	7.33E-05	up
NEU4	2.572029	1.18E-07	1.07E-05	up
ISX	2.571564	2.41E-05	0.000471	up
CLDN1	2.5703	7.26E-07	3.66E-05	up
PLA2G2A	2.557448	8.74E-05	0.001259	up

ABP1	2.539079	7.32E-08	7.77E-06	up
AGMO	2.517833	0.000137	0.001777	up
CA1	2.492693	0.000942	0.007851	up
CHST6	2.489314	3.07E-07	2.01E-05	up
SLC6A19	2.485813	6.04E-05	0.000947	up
GAST	2.483137	0.026779	0.103157	up
ONECUT3	2.471402	3.92E-09	1.43E-06	up
GCG	2.457791	0.001623	0.011972	up
CCL15	2.455748	2.44E-07	1.73E-05	up
GP2	2.438449	1.87E-05	0.00039	up
XLOC_009021	2.43141	1.77E-08	3.11E-06	up
FOXD1	2.426067	1.66E-05	0.000359	up
DEFA5	2.422778	0.010724	0.051601	up
LOC100288092	2.422671	3.69E-08	5.03E-06	up
NAT2	2.417217	1.04E-05	0.000252	up
CDH16	2.416043	8.51E-08	8.59E-06	up
GJB5	2.394721	2.42E-06	8.59E-05	up
NMUR2	2.353981	2.52E-08	3.90E-06	up
APOA4	2.352324	0.019229	0.080616	up
LOC100507056	2.351478	2.68E-06	9.29E-05	up
MALL	2.341713	9.22E-06	0.000229	up
THBS2	2.337974	5.06E-08	6.31E-06	up
LOC100505839	2.333002	3.09E-07	2.01E-05	up
NMU	2.33254	5.38E-08	6.47E-06	up
MLXIPL	2.301372	8.54E-08	8.59E-06	up
MFSD2A	2.292743	1.44E-06	5.96E-05	up
A1CF	2.269594	1.34E-06	5.69E-05	up
C11orf86	2.265592	0.000145	0.001853	up
ASCL2	2.258022	4.14E-08	5.51E-06	up
SHD	2.250606	1.15E-07	1.05E-05	up
DSG3	2.236917	3.45E-05	0.000619	up
NANOS3	2.231927	1.76E-06	7.00E-05	up
NR1I2	2.2289	1.54E-07	1.28E-05	up
LOC100506219	2.221821	5.64E-13	6.86E-09	up
CEACAM3	2.213249	7.58E-07	3.76E-05	up
LOC100124692	2.212013	3.05E-07	2.01E-05	up
TTLL6	2.211426	1.58E-07	1.30E-05	up
KCNJ3	2.201666	2.03E-06	7.60E-05	up
KIAA1257	2.194219	3.05E-09	1.26E-06	up
MARVELD3	2.190204	7.18E-08	7.68E-06	up
SLC6A7	2.16634	6.61E-07	3.44E-05	up
SLC26A3	2.125227	0.003692	0.022668	up
XLOC_005220	2.098529	1.50E-10	2.88E-07	up
RBP4	2.081832	3.17E-06	0.000105	up

ABCG8	2.081098	4.73E-05	0.000789	up
KLK1	2.064512	5.31E-08	6.43E-06	up
CHST5	2.053849	2.07E-06	7.65E-05	up
MTTP	2.04623	0.009486	0.046977	up
REG1B	2.037366	0.001013	0.008304	up
GJB3	2.025351	1.56E-07	1.29E-05	up
OVOL1	2.025147	4.55E-07	2.68E-05	up
INSM1	2.0214	2.71E-06	9.32E-05	up
AGMAT	2.020558	3.16E-07	2.04E-05	up
C19orf45	2.016888	1.90E-10	2.88E-07	up
PRAP1	2.015414	1.74E-05	0.000371	up
MLK7-AS1	2.00665	1.85E-06	7.23E-05	up
CFTR	2.004008	1.46E-06	6.03E-05	up
PCK1	2.000699	0.001532	0.011453	up
ANXA9	1.99741	1.57E-07	1.30E-05	up
PPP1R14D	1.989161	6.16E-06	0.00017	up
XLOC_l2_006021	1.987217	1.53E-05	0.000338	up
FLJ26086	1.984047	2.26E-05	0.000449	up
JPH1	1.979048	6.10E-08	6.91E-06	up
PPP1R14C	1.959387	1.47E-06	6.08E-05	up
FUT5	1.952768	5.28E-08	6.43E-06	up
XLOC_014103	1.94998	1.20E-06	5.22E-05	up
LOC100505908	1.947798	1.03E-08	2.31E-06	up
TIMP4	1.947428	3.67E-09	1.40E-06	up
AFAP1-AS1	1.94618	0.000382	0.003878	up
S100A2	1.940886	4.06E-08	5.42E-06	up
TNFSF9	1.937688	2.27E-06	8.22E-05	up
WISP3	1.937577	7.20E-05	0.001086	up
TNNC2	1.936126	6.49E-07	3.41E-05	up
GAL	1.927642	0.001437	0.0109	up
HOTAIR	1.92246	2.60E-07	1.81E-05	up
TNFRSF11A	1.921	1.25E-06	5.40E-05	up
EPHB2	1.918209	5.31E-08	6.43E-06	up
KRT42P	1.908256	1.35E-06	5.71E-05	up
CCND2	1.905744	1.98E-06	7.45E-05	up
LOC100506995	1.904624	5.69E-06	0.000161	up
CD55	1.904434	1.52E-06	6.22E-05	up
PRR4	1.901341	0.000181	0.002185	up
NOX1	1.900521	5.50E-07	3.03E-05	up
XLOC_007054	1.884718	2.65E-08	4.05E-06	up
NTS	1.878419	7.01E-05	0.001063	up
CNTNAP2	1.873576	3.66E-08	5.03E-06	up
PCSK5	1.871376	1.66E-05	0.00036	up
C2orf82	1.864342	1.09E-08	2.38E-06	up

FAM84A	1.852385	1.00E-08	2.29E-06	up
SULT2B1	1.851737	4.63E-08	5.90E-06	up
TEX11	1.84904	2.78E-07	1.88E-05	up
ATP2C2	1.845756	7.65E-10	7.67E-07	up
UPP1	1.845195	5.40E-07	3.00E-05	up
XLOC_014161	1.839974	4.23E-12	2.26E-08	up
ABHD11-AS1	1.835378	9.81E-07	4.50E-05	up
EPHX4	1.834373	2.56E-05	0.000494	up
ANKS4B	1.833669	2.18E-07	1.62E-05	up
TNFRSF12A	1.83355	2.31E-07	1.68E-05	up
PI3	1.833393	0.008301	0.042468	up
HES2	1.827105	6.64E-06	0.00018	up
FOSB	1.826541	0.000318	0.003371	up
KRT80	1.825608	4.63E-05	0.000774	up
DHRS9	1.822067	2.95E-05	0.00055	up
XLOC_000642	1.820716	5.09E-09	1.60E-06	up
ABCG5	1.816457	0.000166	0.002047	up
ZNF703	1.809515	1.53E-08	2.90E-06	up
BTBD17	1.805966	7.55E-07	3.76E-05	up
MMP3	1.804606	0.001352	0.010413	up
C17orf78	1.799268	0.008476	0.043141	up
KRT16P2	1.796608	1.36E-05	0.000309	up
CCL25	1.794314	0.007879	0.040843	up
LIF	1.794058	3.59E-08	5.03E-06	up
TPPP3	1.790669	1.67E-07	1.35E-05	up
FAM81A	1.788799	1.60E-07	1.31E-05	up
FAM25A	1.788118	0.00013	0.001704	up
ITFG3	1.787497	0.000185	0.002214	up
GBA3	1.787134	0.016459	0.071723	up
SLC5A1	1.782074	1.70E-05	0.000366	up
LOC201651	1.781607	8.52E-06	0.000216	up
TFPI2	1.771952	1.83E-05	0.000384	up
C2orf89	1.767571	1.68E-05	0.000363	up
APOC3	1.767057	0.021661	0.088209	up
XLOC_l2_005553	1.765079	3.36E-07	2.15E-05	up
IGFL2	1.754927	0.000152	0.001918	up
CALHM3	1.75147	0.00039	0.003943	up
XLOC_l2_009947	1.750739	1.18E-05	0.000277	up
ZNF488	1.749951	5.41E-06	0.000156	up
FOLH1B	1.744845	0.001806	0.013042	up
TMEM150B	1.744227	1.53E-07	1.28E-05	up
XDH	1.740048	0.000344	0.003576	up
SLC27A2	1.737823	6.42E-08	7.12E-06	up
XLOC_005777	1.737647	5.03E-05	0.000826	up

XLOC_006948	1.737357	1.01E-07	9.58E-06	up
BHLHE41	1.731726	5.88E-09	1.75E-06	up
HOXB5	1.72908	7.22E-07	3.64E-05	up
SLC7A9	1.725497	0.007874	0.040831	up
LOC100505535	1.721428	6.05E-07	3.24E-05	up
MMP7	1.718529	0.007593	0.039705	up
CRABP2	1.712502	2.78E-05	0.000529	up
ANXA1	1.712011	1.09E-06	4.87E-05	up
SYNPR	1.710974	4.36E-05	0.000738	up
3-Mar	1.705935	3.23E-09	1.29E-06	up
RAB3B	1.695897	5.81E-05	0.000921	up
POLR3G	1.693546	4.33E-06	0.000133	up
KLK12	1.688947	0.000567	0.005257	up
SLC6A14	1.68735	0.002367	0.016084	up
KCNH6	1.684382	1.86E-08	3.21E-06	up
FABP6	1.680514	0.000144	0.001848	up
TNFRSF11B	1.679811	7.13E-06	0.000189	up
CYP3A4	1.671232	0.000851	0.007233	up
PRAC	1.670518	0.000665	0.005972	up
FLJ45248	1.670104	1.90E-07	1.48E-05	up
TBX3	1.66964	6.02E-07	3.23E-05	up
CDHR5	1.666696	5.03E-06	0.000148	up
FOXJ1	1.665203	6.42E-05	0.000992	up
LOC254057	1.660486	0.000291	0.003136	up
XLOC_011893	1.652545	8.66E-10	7.94E-07	up
KLK10	1.651809	4.93E-06	0.000146	up
C14orf176	1.644736	4.57E-05	0.000765	up
CEACAM5	1.631689	0.000111	0.001519	up
PNMT	1.630663	1.67E-08	3.03E-06	up
XLOC_011858	1.629432	1.92E-06	7.37E-05	up
TRIM15	1.629113	2.01E-05	0.000412	up
LINC00346	1.626582	3.16E-08	4.57E-06	up
SLC22A18AS	1.625645	3.13E-07	2.03E-05	up
TRPM2	1.622704	2.67E-07	1.84E-05	up
XLOC_000822	1.618246	3.87E-07	2.35E-05	up
XLOC_012981	1.614681	8.71E-06	0.00022	up
MSX1	1.613899	5.28E-06	0.000153	up
IYD	1.613394	7.60E-09	1.99E-06	up
WBSCR27	1.611348	0.000749	0.00654	up
PPBP	1.611057	0.000186	0.002226	up
CRIP3	1.608697	7.64E-05	0.001139	up
ADRA2A	1.604589	0.000112	0.001536	up
GPRC5A	1.597215	1.76E-06	7.00E-05	up
TTC9B	1.591586	1.51E-05	0.000335	up

C19orf21	1.588836	6.54E-07	3.43E-05	up
HIST3H2A	1.587734	2.53E-06	8.88E-05	up
FAR2	1.581724	1.17E-05	0.000276	up
DNAH2	1.578425	1.78E-05	0.000377	up
XLOC_l2_010149	1.574914	1.42E-06	5.91E-05	up
FEV	1.571827	7.11E-06	0.000189	up
LOC100507218	1.571811	0.000103	0.001433	up
PADI2	1.568478	7.92E-06	0.000205	up
MS4A15	1.568208	0.000106	0.001463	up
CIB2	1.567026	7.50E-07	3.74E-05	up
BTBD16	1.565792	0.000874	0.00737	up
SHISA3	1.563603	0.000784	0.006805	up
NKX6-3	1.562514	8.38E-05	0.001217	up
CCL18	1.557768	0.026874	0.103401	up
SLC16A2	1.553355	9.53E-08	9.18E-06	up
XLOC_l2_001134	1.549668	9.91E-06	0.000242	up
TNS4	1.548133	0.00032	0.003384	up
ETV4	1.545884	1.99E-06	7.48E-05	up
PCSK9	1.544743	0.00402	0.024237	up
UBXN10	1.543238	4.10E-10	5.06E-07	up
WNT11	1.539219	1.30E-06	5.55E-05	up
XLOC_003528	1.538096	0.000457	0.004438	up
CITED1	1.533117	7.08E-07	3.59E-05	up
TPRXL	1.532594	2.93E-05	0.000547	up
KIAA1199	1.531043	0.000333	0.003484	up
BANK1	1.529704	0.000139	0.001801	up
AREG	1.529032	5.05E-06	0.000148	up
NR5A2	1.525026	0.000168	0.00206	up
GCNT3	1.522767	7.85E-05	0.001162	up
NXF3	1.521258	2.60E-05	0.000501	up
GLB1L2	1.519342	2.21E-08	3.58E-06	up
STK31	1.516212	1.07E-06	4.84E-05	up
MEST	1.515007	8.90E-11	2.29E-07	up
TINAG	1.514196	0.00012	0.001618	up
C20orf85	1.511561	0.001272	0.009919	up
CLDN7	1.510021	7.95E-06	0.000205	up
XLOC_007123	1.501849	0.000208	0.002415	up
LDLRAD1	1.501766	5.20E-07	2.94E-05	up
CTSL2	1.501311	2.28E-09	1.17E-06	up
GUCA2A	1.499255	0.002977	0.019217	up
GUCY1B2	1.493444	2.61E-05	0.000503	up
LOC100506591	1.493116	0.000206	0.002396	up
METTL7B	1.48905	1.51E-06	6.19E-05	up
PF4	1.487426	0.000302	0.003235	up

S100A10	1.48369	2.11E-06	7.75E-05	up
XLOC_003441	1.48342	3.02E-08	4.41E-06	up
HP	1.481206	4.25E-06	0.000131	up
XLOC_000095	1.481162	1.67E-07	1.35E-05	up
TUBB3	1.477069	7.41E-05	0.001111	up
ARL9	1.476482	3.45E-05	0.000618	up
PHLDA2	1.472741	5.12E-08	6.31E-06	up
DACH1	1.471617	1.57E-05	0.000344	up
NOXO1	1.471501	1.36E-06	5.74E-05	up
SEMG1	1.468253	0.000233	0.00264	up
KBTBD11	1.467548	5.73E-06	0.000162	up
XLOC_000371	1.467422	0.002033	0.014276	up
CCK	1.46408	0.00184	0.013229	up
C1orf110	1.463335	0.000252	0.002807	up
FOXD2	1.462607	1.81E-08	3.13E-06	up
BEST4	1.459577	0.003355	0.021059	up
HTR2B	1.458794	0.000259	0.002867	up
CXXC4	1.457637	7.41E-08	7.82E-06	up
DAPL1	1.456667	1.49E-05	0.000331	up
ULBP2	1.45457	4.52E-06	0.000136	up
TMPRSS15	1.453955	0.044517	0.150998	up
PDE3B	1.453585	1.98E-07	1.53E-05	up
C6orf97	1.451994	6.67E-06	0.00018	up
MS4A10	1.450186	0.032208	0.118427	up
GPR37L1	1.449708	7.03E-06	0.000187	up
KRT7	1.449367	7.08E-07	3.59E-05	up
AQP2	1.447768	0.000127	0.001677	up
OSTalpha	1.447496	0.026504	0.102307	up
LOC100505882	1.446474	2.52E-06	8.84E-05	up
GLTPD2	1.446179	2.91E-05	0.000544	up
HENMT1	1.445751	4.41E-09	1.52E-06	up
TPPP	1.445247	1.89E-06	7.31E-05	up
ITGB8	1.44495	8.75E-09	2.14E-06	up
HSD11B2	1.441826	2.19E-09	1.17E-06	up
LOC100505592	1.441434	5.69E-05	0.000907	up
XLOC_005540	1.437539	7.37E-07	3.69E-05	up
RNF186	1.437419	0.000522	0.004926	up
LRP8	1.436786	0.000286	0.003094	up
FABP2	1.435683	0.005521	0.030915	up
FUT3	1.435452	2.40E-06	8.56E-05	up
LOC439990	1.42904	3.64E-07	2.27E-05	up
C21orf90	1.428113	0.001382	0.010581	up
MMP12	1.42743	0.029477	0.110694	up
CYP4F2	1.426325	0.00679	0.036395	up

SNAR-A3	1.425288	0.000188	0.002241	up
HIST1H2AI	1.424063	8.16E-06	0.000209	up
ACE2	1.423737	0.004022	0.024237	up
OTOP3	1.422547	0.002122	0.01477	up
SMPX	1.420227	2.04E-05	0.000416	up
CRYBA2	1.419201	6.43E-07	3.40E-05	up
DRD2	1.418903	2.85E-07	1.91E-05	up
CD9	1.415369	9.50E-08	9.18E-06	up
NPNT	1.410604	4.65E-06	0.000139	up
MDFI	1.409329	4.23E-05	0.00072	up
UNC93A	1.407646	9.31E-05	0.001324	up
APOBEC1	1.407533	0.002183	0.015091	up
HPR	1.406461	7.25E-05	0.001091	up
PAX9	1.404133	9.70E-05	0.001366	up
ZP3	1.403601	6.01E-06	0.000167	up
SIPA1L2	1.40314	2.06E-06	7.65E-05	up
DNAJC12	1.401753	1.02E-08	2.29E-06	up
SATB2	1.400351	1.33E-06	5.65E-05	up
KLK3	1.39994	0.000121	0.001625	up
ITLN1	1.398985	0.028017	0.106431	up
LOC170425	1.39792	4.93E-06	0.000146	up
PRSS3	1.396961	2.60E-06	9.06E-05	up
HOXB6	1.396447	0.00013	0.001705	up
HOXB7	1.394688	3.86E-06	0.000122	up
MESP1	1.389641	0.004747	0.027521	up
MUC13	1.383498	0.000539	0.005041	up
B3GNT8	1.383498	6.11E-07	3.25E-05	up
HSPA4L	1.38265	7.74E-05	0.001151	up
MUC2	1.380377	0.004364	0.025809	up
SLC35D3	1.376628	5.61E-06	0.000159	up
AIFM3	1.375639	3.58E-07	2.24E-05	up
SLC4A7	1.374412	0.000149	0.001896	up
PFKFB4	1.371912	5.38E-06	0.000155	up
OR51E1	1.37114	8.11E-05	0.001194	up
C10orf125	1.370611	9.92E-07	4.54E-05	up
LOC100129931	1.365906	3.12E-05	0.000576	up
LOC284865	1.365893	0.000723	0.006359	up
LOC100133311	1.36574	6.51E-06	0.000178	up
SNORA74A	1.365637	0.000181	0.002182	up
CRYM	1.362587	2.43E-07	1.73E-05	up
ABCC2	1.362306	0.002341	0.015974	up
XLOC_005749	1.357892	2.46E-06	8.72E-05	up
XLOC_000348	1.35394	1.93E-06	7.37E-05	up
C10orf81	1.353202	1.76E-06	7.00E-05	up

SNAR-H	1.339955	0.000201	0.002355	up
NEUROD2	1.339429	0.000912	0.007632	up
MLIP	1.339362	0.000329	0.003462	up
PCDHB9	1.337358	3.89E-05	0.000675	up
XLOC_l2_010056	1.335785	6.57E-06	0.000179	up
XLOC_l2_005814	1.334971	1.49E-05	0.000331	up
LOC100190940	1.331275	3.01E-06	0.000101	up
SPAG17	1.330809	0.003232	0.020456	up
CDSN	1.328778	8.06E-05	0.001189	up
BAIAP2L2	1.328314	0.000198	0.00233	up
DPEP1	1.325917	0.028642	0.108266	up
RRM2	1.325131	0.000444	0.004349	up
LGR5	1.322958	2.89E-05	0.000542	up
RAD51AP1	1.321439	4.39E-05	0.000742	up
LYPD5	1.320305	6.13E-05	0.000958	up
SP6	1.320079	2.79E-06	9.51E-05	up
TUBAL3	1.318096	0.002947	0.019075	up
CASP14L	1.317237	0.003703	0.022708	up
CREB3L3	1.316176	0.006357	0.03451	up
EPPK1	1.315071	2.21E-06	8.04E-05	up
HSD17B11	1.312542	4.99E-07	2.86E-05	up
AGR3	1.311365	1.28E-07	1.12E-05	up
ITLN2	1.308596	0.032615	0.119579	up
MFI2	1.308337	1.03E-05	0.000249	up
C6orf208	1.30668	1.27E-06	5.45E-05	up
NUP62CL	1.306516	1.94E-06	7.40E-05	up
F5	1.306156	0.000899	0.00754	up
DEPDC1B	1.302553	6.00E-05	0.000942	up
MACC1	1.300405	1.65E-07	1.34E-05	up
CDA	1.299433	0.000686	0.006121	up
S100A3	1.298853	0.00045	0.004384	up
KIAA1211	1.296637	0.000107	0.001475	up
ARHGEF38	1.295017	6.14E-08	6.91E-06	up
PTPRVP	1.2945	0.003027	0.019454	up
RCOR2	1.294114	5.22E-09	1.62E-06	up
ETS2	1.29273	2.29E-08	3.64E-06	up
HTR1D	1.292704	2.39E-05	0.000469	up
CELSR3	1.289163	2.68E-06	9.28E-05	up
FABP1	1.288601	0.013214	0.060547	up
LOC100507235	1.287655	4.96E-06	0.000146	up
DHFR	1.287615	2.14E-07	1.61E-05	up
ACTL8	1.287365	0.000607	0.005554	up
SNAR-D	1.286848	0.000403	0.004038	up
XPNPEP2	1.28556	0.000867	0.007321	up

PRR19	1.285082	1.28E-08	2.64E-06	up
CALML4	1.284737	2.06E-06	7.65E-05	up
SNAR-G2	1.283926	0.00046	0.004462	up
XLOC_l2_007986	1.283471	0.000384	0.003894	up
PYDC1	1.28318	6.19E-06	0.000171	up
SH3BP4	1.27952	1.33E-09	9.69E-07	up
LRRIQ4	1.279331	4.38E-05	0.00074	up
MGC12982	1.276727	2.91E-07	1.94E-05	up
TLX1	1.276552	3.39E-07	2.16E-05	up
XLOC_005341	1.276225	0.007079	0.037567	up
AFAP1L2	1.275236	1.98E-06	7.45E-05	up
MYOM3	1.274445	3.17E-05	0.000582	up
ANXA2P3	1.270675	5.09E-05	0.000835	up
XLOC_002921	1.269621	1.11E-06	4.93E-05	up
XLOC_l2_006745	1.268796	8.05E-06	0.000207	up
CYP1A1	1.267997	0.005011	0.028685	up
XLOC_001341	1.265884	8.26E-05	0.001207	up
FAM64A	1.265584	4.11E-05	0.000706	up
LOC93432	1.264064	1.42E-05	0.000319	up
KRT6B	1.263704	0.020106	0.083396	up
CEACAM7	1.260476	0.000321	0.003394	up
OR7E5P	1.260424	5.27E-05	0.000856	up
LRRC6	1.257069	8.73E-07	4.17E-05	up
HKDC1	1.255298	4.86E-05	0.000806	up
ITPK1	1.254585	3.83E-06	0.000121	up
CEACAM1	1.252966	0.00015	0.001899	up
MYO15B	1.249643	2.85E-05	0.000538	up
LOC100507507	1.247935	3.13E-09	1.27E-06	up
LOC648987	1.246861	2.10E-06	7.73E-05	up
CTTNBP2	1.245904	5.88E-05	0.000929	up
C1orf135	1.24442	5.17E-05	0.000844	up
SKAP2	1.243282	7.38E-09	1.99E-06	up
TMC7	1.242899	7.64E-06	0.000199	up
SKA1	1.242619	0.000158	0.001976	up
CENPA	1.241323	0.000203	0.002375	up
EGR4	1.239898	6.65E-06	0.00018	up
INSL5	1.237019	0.000996	0.008185	up
ETHE1	1.234881	8.05E-07	3.93E-05	up
XLOC_l2_015800	1.231556	0.008937	0.044926	up
MEP1B	1.230226	0.037193	0.132311	up
C3orf32	1.229688	0.001188	0.009418	up
LOC389641	1.229391	3.41E-07	2.17E-05	up
SKP2	1.223535	1.26E-07	1.12E-05	up
CST1	1.222913	1.06E-05	0.000256	up

GPD1	1.222563	0.000414	0.004127	up
GJB2	1.219959	1.60E-05	0.00035	up
ANLN	1.217399	0.000321	0.003394	up
XLOC_002870	1.21226	0.000368	0.003774	up
TMEM45B	1.212124	3.04E-07	2.01E-05	up
CDHR4	1.211703	0.000333	0.003484	up
KIF13A	1.210303	9.56E-06	0.000236	up
KRT15	1.209533	2.56E-07	1.78E-05	up
LOC284570	1.208332	6.07E-06	0.000169	up
FOXC1	1.207511	6.21E-05	0.000968	up
ODZ2	1.205905	6.92E-05	0.001053	up
LOC100287482	1.204302	3.38E-05	0.00061	up
LOC100505644	1.203251	2.77E-06	9.45E-05	up
LOC401127	1.203119	3.75E-07	2.31E-05	up
NEB	1.202715	0.000386	0.003913	up
PHOSPHO2-KLHL23	1.202069	9.85E-05	0.001385	up
XLOC_006544	1.200701	1.58E-08	2.93E-06	up
VGF	1.197867	0.004658	0.027099	up
C18orf56	1.196397	1.58E-05	0.000346	up
ASS1	1.195619	1.06E-05	0.000256	up
ABHD11	1.194861	4.36E-08	5.66E-06	up
MNS1	1.194754	0.000163	0.002019	up
SNAR-B2	1.194278	0.000995	0.008182	up
ANXA2	1.19299	7.11E-05	0.001074	up
GDA	1.190203	3.12E-05	0.000577	up
EDN3	1.19009	9.53E-06	0.000235	up
XLOC_006705	1.187874	3.95E-05	0.000685	up
XLOC_012170	1.187026	2.75E-07	1.87E-05	up
SLC7A4	1.18694	0.001362	0.01047	up
TNNC1	1.186188	0.002398	0.01626	up
MS4A8B	1.183968	2.77E-05	0.000527	up
LOC285419	1.183205	0.000104	0.001442	up
XLOC_000855	1.182012	5.65E-06	0.00016	up
EPHB1	1.181156	5.02E-06	0.000148	up
LOC100505727	1.180853	0.000219	0.002507	up
XLOC_008005	1.179488	0.000102	0.001425	up
CES2	1.178783	4.16E-05	0.000713	up
RIMKLA	1.178051	1.57E-05	0.000344	up
GRB14	1.177615	0.000205	0.002382	up
CDKN3	1.177073	3.43E-05	0.000618	up
BST1	1.176709	0.000563	0.005238	up
KCNH8	1.174498	4.36E-07	2.59E-05	up
C11orf80	1.174364	4.52E-06	0.000136	up

XLOC_003245	1.173682	0.00017	0.002083	up
LOC388242	1.172984	6.75E-05	0.001033	up
SKA3	1.171853	4.92E-05	0.000812	up
C2CD4A	1.170766	0.001132	0.009056	up
C12orf28	1.170759	0.000633	0.005745	up
REEP6	1.169646	8.07E-06	0.000207	up
IRAK2	1.168946	3.13E-05	0.000578	up
GALNT8	1.168197	0.001122	0.008991	up
KLHL13	1.167455	0.000455	0.004418	up
SPC25	1.165473	0.000423	0.004197	up
ENTPD2	1.163585	2.07E-07	1.57E-05	up
PBK	1.163469	0.000289	0.003118	up
AGXT	1.162437	3.08E-05	0.000571	up
CENPH	1.161537	4.19E-05	0.000716	up
TMEM158	1.159797	0.000217	0.002494	up
TFAP2C	1.156589	2.70E-07	1.85E-05	up
PKDCC	1.154594	4.28E-05	0.000727	up
KCNE3	1.154086	7.36E-06	0.000194	up
NOL4	1.153824	0.000569	0.005271	up
FAM72A	1.153168	0.000428	0.004231	up
PRSS2	1.14976	6.78E-06	0.000183	up
XLOC_l2_014101	1.149759	8.62E-08	8.62E-06	up
PPFIA3	1.146131	1.07E-05	0.000257	up
SNAR-F	1.14564	0.001651	0.01214	up
XLOC_002867	1.143406	3.67E-06	0.000117	up
ARSE	1.141604	8.54E-05	0.001238	up
PITX2	1.141523	0.001279	0.009956	up
ZNF462	1.139313	6.73E-08	7.37E-06	up
LOC283663	1.138654	0.000176	0.002138	up
MATN2	1.137159	6.59E-06	0.000179	up
NT5E	1.136819	8.25E-05	0.001206	up
SLC6A20	1.136117	0.001154	0.009205	up
ACRBP	1.132736	2.66E-06	9.26E-05	up
EPHA1	1.132239	8.04E-07	3.93E-05	up
VIPR1	1.132107	9.35E-07	4.37E-05	up
CXCL3	1.132099	0.015069	0.066986	up
XLOC_003355	1.130686	3.06E-06	0.000102	up
ESCO2	1.130426	1.55E-05	0.00034	up
CLSPN	1.129052	0.000117	0.001586	up
FAM176A	1.128928	3.13E-05	0.000578	up
MESP2	1.128301	9.53E-06	0.000235	up
SCG2	1.126428	0.003378	0.021168	up
CDC6	1.125782	0.000182	0.002194	up
LOC388780	1.125709	0.003053	0.019585	up

TM6SF2	1.125557	0.000728	0.006394	up
TPMT	1.122442	2.28E-08	3.64E-06	up
PVRL1	1.121782	2.08E-06	7.69E-05	up
LOC100506957	1.12167	0.002703	0.017824	up
ACOX2	1.119954	1.84E-05	0.000385	up
CDHR2	1.117899	0.000996	0.008185	up
CCNA2	1.117389	0.000296	0.00318	up
EFNA3	1.116108	4.89E-05	0.000809	up
FAM72D	1.116066	6.29E-05	0.000976	up
XLOC_013754	1.115233	4.10E-05	0.000705	up
LMTK3	1.115147	1.06E-06	4.79E-05	up
PRR5L	1.113174	8.41E-06	0.000214	up
CLCN2	1.112632	1.03E-06	4.69E-05	up
QPCT	1.111537	0.000146	0.001865	up
MANEAL	1.108842	6.44E-09	1.83E-06	up
S100A6	1.108096	4.18E-07	2.51E-05	up
PDSS1	1.107798	2.50E-05	0.000485	up
HIST1H2BF	1.1064	0.000123	0.00164	up
TRIM31	1.104944	0.000286	0.00309	up
LOC100128593	1.103215	5.55E-06	0.000158	up
FLVCR2	1.103124	0.001622	0.011968	up
XLOC_011755	1.101837	6.65E-07	3.46E-05	up
IL22RA1	1.101263	9.28E-06	0.00023	up
SSX2IP	1.101222	6.15E-06	0.00017	up
PLK4	1.100761	7.36E-05	0.001105	up
YBX2	1.100742	0.001776	0.012874	up
XLOC_003735	1.100701	3.98E-05	0.000687	up
LOC389634	1.099796	0.000224	0.002547	up
ZIC5	1.099713	6.33E-05	0.00098	up
SHCBP1	1.09927	0.001803	0.013029	up
TGM2	1.097379	0.006204	0.033846	up
C9orf40	1.096991	2.63E-07	1.83E-05	up
CKAP2L	1.096902	0.000462	0.004471	up
ABCC13	1.095574	0.033434	0.12176	up
XLOC_012977	1.09529	0.000273	0.002981	up
NEK11	1.094245	2.57E-06	8.99E-05	up
FAM151B	1.093573	1.31E-05	0.0003	up
CEP55	1.092864	0.000325	0.003425	up
NR1H4	1.090447	0.021889	0.088789	up
FLJ39095	1.090393	8.00E-05	0.001183	up
GPR153	1.089357	0.001018	0.008334	up
PITX1	1.088165	2.35E-06	8.45E-05	up
ANXA8L2	1.087666	0.000796	0.006885	up
LIPG	1.087491	0.000902	0.007556	up

MYBL2	1.084013	0.000464	0.004492	up
CEACAM20	1.083633	3.55E-06	0.000115	up
LOC100287415	1.083627	0.000357	0.003679	up
MAD2L1	1.08212	0.000211	0.002443	up
CXCL1	1.081704	0.013536	0.061663	up
RAD54L	1.080709	0.00015	0.0019	up
LOC100505909	1.080438	0.000686	0.00612	up
POLE2	1.079749	2.18E-05	0.000437	up
FLJ35424	1.079348	0.042897	0.14707	up
BDKRB1	1.079209	9.44E-05	0.001339	up
LINC00336	1.078609	3.51E-05	0.000626	up
XLOC_009181	1.078305	0.000975	0.00806	up
KRT16	1.077474	0.004451	0.026193	up
KIFC1	1.077466	0.000219	0.002507	up
GDPD2	1.076644	0.001563	0.011645	up
ARX	1.076215	2.15E-05	0.000432	up
NOD1	1.075657	5.20E-06	0.000151	up
FAM131B	1.075531	0.000162	0.002014	up
DYX1C1	1.07474	8.85E-06	0.000222	up
TMPRSS4	1.074259	7.69E-06	0.0002	up
ABTB2	1.074144	0.000114	0.001552	up
HSD17B2	1.074061	0.000277	0.003021	up
CST6	1.070371	0.023205	0.092956	up
CYP4F3	1.070087	0.006357	0.03451	up
INPP1	1.069755	9.19E-06	0.000229	up
FAM54A	1.069687	0.000127	0.001677	up
LCN15	1.068965	0.006056	0.033234	up
LOC100134138	1.0686	0.014233	0.064116	up
SLC6A12	1.068207	0.034178	0.123809	up
LDHC	1.067511	0.001032	0.008427	up
CYP27B1	1.067406	4.45E-06	0.000135	up
XLOC_014263	1.065577	0.001045	0.008497	up
XLOC_l2_008289	1.065433	2.70E-06	9.32E-05	up
MTMR11	1.064886	6.29E-05	0.000977	up
MSX2P1	1.063877	7.05E-07	3.59E-05	up
TM4SF1	1.062646	1.55E-06	6.30E-05	up
DHRS11	1.062493	0.000132	0.001728	up
XLOC_005924	1.062085	2.73E-06	9.34E-05	up
XLOC_l2_013480	1.060808	5.51E-06	0.000158	up
ACAA2	1.060064	6.60E-07	3.44E-05	up
TMEM139	1.058597	1.13E-05	0.000268	up
XLOC_007398	1.058447	0.010951	0.052445	up
TRIM7	1.058441	0.003277	0.020678	up
TFPI	1.057876	0.000106	0.001468	up

BCMO1	1.057792	0.001218	0.009598	up
SLC18A1	1.057688	7.72E-05	0.001149	up
IL11	1.056882	0.001277	0.009946	up
SERPINB8	1.056208	0.000339	0.003532	up
PTPRO	1.055077	0.009474	0.046936	up
MPV17L	1.053554	1.72E-05	0.000369	up
PLCB3	1.052388	9.59E-06	0.000236	up
AQP12A	1.052341	0.00029	0.003128	up
SNORD86	1.051101	0.000105	0.001448	up
FAS	1.048982	2.04E-05	0.000416	up
P2RX6P	1.048443	0.000601	0.005514	up
KREMEN2	1.048146	0.000882	0.007431	up
KHK	1.048071	0.001573	0.011696	up
GPR172B	1.047663	0.005221	0.0296	up
RAD51	1.047506	0.00013	0.001703	up
CENPK	1.046856	0.000434	0.004278	up
SLC35G1	1.045094	0.00561	0.031315	up
TIMP1	1.044383	4.17E-08	5.51E-06	up
C11orf82	1.044356	0.001525	0.011419	up
CA13	1.044158	1.56E-05	0.000343	up
MCM10	1.043581	0.001357	0.010445	up
E2F2	1.04092	0.000141	0.001816	up
HIST1H3J	1.040832	6.76E-06	0.000182	up
TFAP2A	1.040236	4.90E-05	0.000811	up
C20orf202	1.0385	1.31E-05	0.0003	up
C9orf171	1.037533	0.000298	0.003192	up
XLOC_008426	1.037481	0.004688	0.02724	up
PROM1	1.036397	7.16E-06	0.00019	up
ACER3	1.036289	5.64E-05	0.0009	up
PRR7	1.036002	5.74E-07	3.10E-05	up
OIP5	1.035668	0.000226	0.002573	up
CCDC68	1.035572	0.000682	0.006091	up
XLOC_l2_008546	1.035561	1.18E-07	1.07E-05	up
HOXB3	1.03503	6.26E-05	0.000974	up
HIST1H2AG	1.03275	0.000114	0.001551	up
XLOC_009398	1.032687	0.013277	0.060762	up
LOC100288911	1.031857	1.96E-05	0.000404	up
ADRA2C	1.031275	5.54E-05	0.000891	up
P2RY2	1.03029	0.000969	0.008016	up
LOC100506110	1.029508	9.78E-07	4.50E-05	up
MAGEA2B	1.027608	0.011548	0.0547	up
LGALS4	1.027255	1.42E-05	0.000318	up
C1QTNF1	1.024717	0.004671	0.02716	up
ACSBG1	1.024662	0.002512	0.016807	up

MOCOS	1.023867	8.93E-05	0.001278	up
KIF12	1.023756	4.61E-09	1.56E-06	up
TSPAN2	1.023555	0.000131	0.00171	up
PLBD1	1.023234	1.75E-08	3.10E-06	up
EPCAM	1.021512	2.54E-07	1.78E-05	up
MYT1	1.020803	9.25E-07	4.34E-05	up
MAGEA6	1.020324	0.0253	0.098993	up
XLOC_007093	1.020081	5.47E-07	3.02E-05	up
KIAA1462	1.0192	0.002678	0.0177	up
DUSP10	1.018215	4.86E-07	2.81E-05	up
GSG2	1.017339	0.00104	0.008474	up
HIST1H1B	1.016787	0.000168	0.002061	up
KDM1B	1.016616	3.31E-06	0.000109	up
ARSJ	1.016097	9.96E-05	0.001397	up
MSMP	1.014972	3.77E-06	0.00012	up
LOC647086	1.014779	2.32E-05	0.000458	up
NEK2	1.014767	0.000251	0.002799	up
GPSM2	1.014465	6.49E-07	3.41E-05	up
HMOX1	1.014306	0.00024	0.002706	up
CDCA2	1.012732	0.000969	0.008016	up
SLC38A11	1.012076	0.002672	0.017669	up
F3	1.011991	6.83E-06	0.000184	up
CYP3A7	1.011546	0.002089	0.014593	up
XLOC_006200	1.011218	0.006542	0.035324	up
RDM1	1.010632	0.000958	0.007945	up
RSPH1	1.010469	1.11E-07	1.02E-05	up
LOC100506561	1.00645	0.000126	0.001669	up
NR6A1	1.005645	1.24E-05	0.000287	up
SLC46A3	1.005473	0.005355	0.030209	up
INPP4A	1.005126	1.06E-05	0.000256	up
PODNL1	1.005064	9.58E-07	4.43E-05	up
MEX3A	1.003529	0.001082	0.008722	up
XLOC_012638	1.002299	0.001863	0.013356	up
PCDHB14	1.002245	0.001315	0.010183	up
HIST2H3A	1.001526	0.000236	0.002666	up
GALM	1.001318	1.47E-07	1.25E-05	up
C1orf201	1.000218	1.82E-06	7.17E-05	up
NEFL	-1.00057	0.000962	0.007972	down
COBLL1	-1.00129	7.46E-08	7.84E-06	down
XLOC_009945	-1.00135	0.000376	0.003834	down
ATP7A	-1.00163	3.40E-06	0.000111	down
XLOC_014068	-1.0035	0.000389	0.003932	down
XLOC_009303	-1.00379	0.014511	0.065026	down
SEMA4A	-1.00453	9.33E-08	9.11E-06	down

XLOC_005101	-1.00475	4.49E-05	0.000755	down
PLVAP	-1.00514	1.83E-05	0.000385	down
LOC100294145	-1.00559	7.13E-05	0.001077	down
WTIP	-1.00568	1.38E-06	5.82E-05	down
RGS11	-1.00599	0.000856	0.007252	down
TM7SF2	-1.00655	7.35E-06	0.000194	down
PRKD1	-1.00675	2.17E-05	0.000435	down
XLOC_001441	-1.00683	0.002137	0.014854	down
XLOC_010308	-1.00767	0.000204	0.002377	down
HLA-DQA1	-1.00797	0.020379	0.08419	down
DBN1	-1.00908	0.000505	0.004805	down
MIR143HG	-1.00955	0.000253	0.002816	down
XLOC_001641	-1.00958	8.34E-05	0.001214	down
FBXO17	-1.01072	3.07E-05	0.000569	down
CHI3L2	-1.01151	0.004305	0.02556	down
LOC285191	-1.01241	8.54E-05	0.001238	down
PSPN	-1.01325	0.000138	0.001793	down
AMHR2	-1.0133	0.000496	0.004733	down
CCDC169	-1.01391	4.40E-05	0.000743	down
SLC9A9	-1.01465	1.03E-06	4.68E-05	down
GPR68	-1.01517	3.86E-09	1.43E-06	down
XLOC_008183	-1.01564	1.60E-07	1.31E-05	down
TBC1D10C	-1.01569	8.67E-07	4.16E-05	down
XLOC_002953	-1.01576	6.72E-05	0.00103	down
SOX2	-1.01682	0.000234	0.002655	down
ADAMTS19	-1.01835	0.00081	0.006969	down
C15orf27	-1.01836	0.002205	0.015219	down
KCNQ1	-1.01847	0.00022	0.002517	down
SLC6A16	-1.0203	4.33E-07	2.58E-05	down
XLOC_001066	-1.02056	0.002058	0.014421	down
IGFBP2	-1.02101	9.69E-07	4.47E-05	down
LOC100507673	-1.02104	7.64E-05	0.001139	down
DOCK2	-1.02113	1.29E-05	0.000297	down
XLOC_012083	-1.02132	0.002494	0.016715	down
DPYSL4	-1.02339	2.67E-05	0.000511	down
GTF3C3	-1.02378	2.38E-05	0.000466	down
CMAHP	-1.02416	2.72E-06	9.32E-05	down
TNFRSF8	-1.02474	0.000421	0.004178	down
LRRC27	-1.02645	1.74E-05	0.000371	down
MAF	-1.02755	0.000177	0.002144	down
GPRC5C	-1.02857	5.74E-06	0.000162	down
LPL	-1.02934	0.004284	0.025463	down
MDGA1	-1.02969	0.000613	0.005596	down
XLOC_009023	-1.03037	1.61E-05	0.000352	down

GZMK	-1.03064	0.000324	0.003417	down
TRIM53P	-1.03073	0.000335	0.003497	down
LOC401847	-1.03074	0.001799	0.013011	down
SLC22A12	-1.03148	6.56E-05	0.00101	down
XLOC_006916	-1.03319	0.00038	0.003868	down
GSTA2	-1.03459	0.002297	0.015717	down
XLOC_l2_004840	-1.03525	8.14E-05	0.001196	down
GJD3	-1.03558	1.82E-06	7.15E-05	down
FAM183A	-1.03596	0.000738	0.006457	down
PSTPIP1	-1.0362	1.04E-07	9.74E-06	down
RNASE1	-1.03805	0.000182	0.002191	down
XLOC_l2_011908	-1.03858	0.000192	0.002287	down
TMEM121	-1.03872	1.36E-06	5.74E-05	down
SMAD9	-1.03878	8.75E-07	4.17E-05	down
LOC100652948	-1.039	6.99E-05	0.001061	down
MEI1	-1.03982	0.000344	0.00357	down
MLL3	-1.03995	5.38E-07	3.00E-05	down
SGK1	-1.04028	0.00021	0.002435	down
ACSM5	-1.04034	1.86E-05	0.000388	down
GSTA5	-1.04093	0.000986	0.008134	down
XLOC_l2_015894	-1.04233	1.98E-06	7.45E-05	down
SALL2	-1.04258	2.85E-07	1.91E-05	down
CFH	-1.04283	0.000129	0.001692	down
OR4D11	-1.0429	0.001771	0.012848	down
XLOC_013965	-1.04357	6.72E-07	3.48E-05	down
C1orf190	-1.04495	4.04E-05	0.000697	down
LPHN2	-1.04526	3.69E-06	0.000117	down
LOC100507333	-1.04698	1.69E-05	0.000366	down
SPOCK1	-1.04771	0.000239	0.002694	down
IL7R	-1.04888	6.83E-05	0.001043	down
PRKCB	-1.04906	0.000504	0.0048	down
LONRF2	-1.04944	0.004152	0.024839	down
XLOC_000712	-1.04977	1.20E-05	0.000279	down
ZNF831	-1.05046	0.000112	0.001537	down
DNAH8	-1.05205	0.000185	0.002217	down
XLOC_l2_013863	-1.05206	8.12E-06	0.000208	down
PLA2G1B	-1.0526	0.01579	0.069382	down
XLOC_000643	-1.05362	0.003395	0.021245	down
LOC100130345	-1.05389	2.85E-05	0.000538	down
FAM127C	-1.05529	0.000183	0.002203	down
METTL7A	-1.05718	3.85E-07	2.34E-05	down
GAS1	-1.05748	0.000324	0.003417	down
LOC100507309	-1.05947	3.35E-06	0.00011	down
LOC283075	-1.05962	0.000265	0.002916	down

XLOC_005548	-1.05997	5.75E-05	0.000914	down
ME3	-1.06065	4.38E-07	2.60E-05	down
FCHSD2	-1.06124	4.40E-10	5.13E-07	down
MID2	-1.06181	3.50E-05	0.000625	down
XLOC_000978	-1.06265	5.62E-06	0.00016	down
SIGLEC1	-1.06342	1.05E-05	0.000253	down
XLOC_l2_012855	-1.06366	6.30E-05	0.000977	down
FLJ37644	-1.06375	0.001857	0.013319	down
KL	-1.06419	9.70E-05	0.001366	down
BEAN1	-1.0653	0.000436	0.004288	down
ST3GAL6	-1.06531	9.62E-06	0.000237	down
FCRL2	-1.07018	0.002346	0.015993	down
OXCT1	-1.07052	9.82E-08	9.35E-06	down
XLOC_011023	-1.07073	0.000647	0.005851	down
PLTP	-1.07186	0.000177	0.002147	down
CCDC106	-1.07259	6.67E-06	0.00018	down
XLOC_l2_005415	-1.07358	1.31E-05	0.0003	down
ADAM28	-1.07374	1.82E-05	0.000384	down
LOC93444	-1.07525	8.33E-05	0.001214	down
C20orf194	-1.07595	3.35E-07	2.15E-05	down
ATG9B	-1.07872	0.002364	0.016079	down
DAND5	-1.07882	0.001995	0.014086	down
OBSL1	-1.07912	6.47E-07	3.41E-05	down
ZNF512B	-1.07931	5.55E-07	3.04E-05	down
SLC22A10	-1.07997	0.000307	0.003277	down
XLOC_004283	-1.08	0.000245	0.002743	down
GOLGA8A	-1.08013	5.94E-05	0.000936	down
LOC728723	-1.08157	8.81E-05	0.001266	down
Q6ILE4	-1.08241	1.14E-05	0.00027	down
ARMCX2	-1.08264	2.04E-07	1.55E-05	down
TESC	-1.0832	0.000164	0.002025	down
MRAS	-1.08501	1.74E-06	6.97E-05	down
SLC4A2	-1.08643	2.95E-06	9.94E-05	down
CCR10	-1.08659	0.000237	0.002678	down
SNORA48	-1.0866	2.78E-05	0.000528	down
TNFRSF13C	-1.08708	0.000262	0.002886	down
CCDC101	-1.08773	0.00011	0.001515	down
HS6ST3	-1.08833	0.000115	0.001564	down
XLOC_003872	-1.08895	0.00026	0.002871	down
NIN	-1.08908	2.96E-07	1.96E-05	down
DMRTC1	-1.08984	0.000354	0.003661	down
LOC100506662	-1.09001	0.000217	0.002492	down
LOC728503	-1.09082	8.29E-05	0.001209	down
XLOC_012145	-1.09234	2.84E-05	0.000537	down

SLC9A3	-1.0925	5.59E-05	0.000897	down
CAPN13	-1.09303	5.62E-07	3.06E-05	down
ITIH4	-1.09447	4.79E-07	2.78E-05	down
XLOC_l2_006138	-1.09458	0.00189	0.013501	down
XLOC_006793	-1.09486	0.005948	0.032738	down
VWA5B1	-1.09499	1.35E-05	0.000308	down
SARS	-1.09521	8.68E-07	4.16E-05	down
GPX3	-1.0965	0.000484	0.004644	down
NR3C1	-1.09651	2.31E-05	0.000457	down
ATP1B2	-1.09691	1.18E-05	0.000277	down
XLOC_l2_008343	-1.09827	6.27E-05	0.000975	down
RASAL3	-1.0987	1.59E-09	1.04E-06	down
GHR	-1.09903	2.14E-06	7.81E-05	down
XLOC_008925	-1.09908	0.005524	0.030926	down
INE1	-1.09944	3.68E-05	0.000648	down
RBP7	-1.09947	3.33E-05	0.000605	down
LOC100292909	-1.10011	3.24E-06	0.000107	down
XLOC_011088	-1.10032	4.73E-05	0.000789	down
NYX	-1.10106	6.07E-07	3.24E-05	down
FAM46C	-1.10168	7.44E-09	1.99E-06	down
CD79B	-1.10215	0.000949	0.007889	down
KLHL6	-1.10236	0.000212	0.00245	down
DCLK2	-1.10461	5.88E-06	0.000165	down
OR56B1	-1.10502	0.000712	0.006286	down
LOC200261	-1.10505	0.000193	0.00229	down
NUP210	-1.10512	0.000196	0.002313	down
C1orf130	-1.10535	0.000495	0.004727	down
SIGLEC14	-1.1062	8.65E-06	0.000219	down
ABCA6	-1.10719	4.81E-07	2.79E-05	down
LOC100130093	-1.10719	6.82E-07	3.52E-05	down
HLA-DOA	-1.10896	0.000173	0.002109	down
XLOC_l2_010330	-1.10924	0.000108	0.001491	down
OPN1MW	-1.1095	0.000994	0.008182	down
MMP19	-1.11171	2.14E-06	7.81E-05	down
STOX2	-1.11225	2.84E-07	1.91E-05	down
IGJ	-1.11409	3.11E-05	0.000576	down
XLOC_002411	-1.11419	4.51E-06	0.000136	down
NES	-1.11441	1.10E-06	4.92E-05	down
FAM20C	-1.11484	9.34E-08	9.11E-06	down
TRPV6	-1.11508	2.89E-05	0.000542	down
GLIS2	-1.1185	2.38E-06	8.50E-05	down
WNT6	-1.1189	0.000171	0.00209	down
S1PR3	-1.12037	8.89E-05	0.001274	down
WIPF3	-1.12434	5.20E-07	2.94E-05	down

P2RY8	-1.12445	7.20E-06	0.00019	down
XLOC_007700	-1.12529	2.36E-05	0.000465	down
LOC729178	-1.12532	0.000274	0.002993	down
NGFRAP1	-1.12754	6.94E-06	0.000186	down
PELI2	-1.12805	2.09E-06	7.73E-05	down
SLITRK4	-1.12974	0.00043	0.004244	down
OPN4	-1.13019	0.000172	0.002101	down
LOC100507334	-1.13114	0.00038	0.003862	down
NRTN	-1.1331	0.001251	0.009788	down
VPREB3	-1.13328	0.000946	0.007871	down
SPIRE1	-1.13351	3.30E-08	4.74E-06	down
LOC401317	-1.13508	3.56E-05	0.000631	down
XLOC_010065	-1.1351	3.04E-05	0.000564	down
FAM155B	-1.13592	0.004256	0.025323	down
CA4	-1.13595	0.000506	0.004807	down
RIMBP3	-1.13655	1.96E-07	1.52E-05	down
TCL1A	-1.14075	0.038842	0.136709	down
LOC100128398	-1.14088	1.23E-05	0.000286	down
JMJD5	-1.14152	3.29E-07	2.12E-05	down
AIM2	-1.14307	0.002859	0.01863	down
LDLRAD2	-1.14374	0.000222	0.002536	down
FAM161A	-1.14411	9.68E-06	0.000238	down
FST	-1.14451	0.003911	0.0237	down
ST8SIA5	-1.14499	0.00017	0.00208	down
PKNOX2	-1.14573	5.75E-05	0.000914	down
KCNMB2	-1.14621	0.004407	0.025997	down
CHGA	-1.14622	0.00041	0.004095	down
CD27	-1.1464	6.81E-06	0.000183	down
DKFZP761C1711	-1.14676	3.23E-05	0.000589	down
ANKRD29	-1.14792	2.17E-05	0.000435	down
CSMD3	-1.14993	0.000164	0.002027	down
NAPSA	-1.15215	1.12E-08	2.42E-06	down
CNTN3	-1.15308	8.85E-05	0.001271	down
ZNF300P1	-1.15338	0.000154	0.001934	down
SLC18A2	-1.15455	1.79E-05	0.000379	down
TLE4	-1.15761	0.000607	0.005554	down
HLA-DOB	-1.1594	0.000467	0.004515	down
TGFB1I1	-1.16099	9.10E-06	0.000227	down
RNF152	-1.16177	0.004354	0.025761	down
SBK2	-1.162	0.000141	0.001821	down
C8orf46	-1.16239	7.77E-07	3.83E-05	down
ADCY1	-1.16485	0.000126	0.001666	down
NDN	-1.16571	2.69E-07	1.85E-05	down
PKIB	-1.1672	9.65E-06	0.000237	down

XLOC_l2_000920	-1.16756	1.01E-05	0.000245	down
APOA5	-1.16846	0.000918	0.007681	down
XLOC_010998	-1.16895	4.77E-05	0.000794	down
RAPSN	-1.16938	0.000166	0.002049	down
IRAK3	-1.17097	0.000173	0.002103	down
CFC1	-1.17628	0.000251	0.002799	down
FGF13	-1.17681	0.000163	0.002016	down
LOC100506123	-1.17727	0.000734	0.006436	down
TDRD9	-1.17786	0.005984	0.032896	down
ATXN7L2	-1.17932	8.32E-05	0.001212	down
BRD4	-1.18064	5.55E-07	3.04E-05	down
ZDBF2	-1.18183	8.70E-06	0.00022	down
CLECL1	-1.18462	0.000524	0.004938	down
LOC100507149	-1.18487	3.96E-05	0.000686	down
RIC3	-1.18541	3.35E-06	0.00011	down
XLOC_003603	-1.18561	2.13E-05	0.00043	down
APOE	-1.18688	5.52E-06	0.000158	down
ALDH1A1	-1.1877	6.58E-08	7.23E-06	down
TMEM235	-1.18881	0.000171	0.002094	down
EFS	-1.18944	5.87E-07	3.16E-05	down
MCHR1	-1.18947	6.66E-05	0.001022	down
ZBTB16	-1.19177	0.000115	0.001565	down
XLOC_003062	-1.1923	9.75E-05	0.001372	down
TCTN2	-1.19232	0.000934	0.007793	down
MAFG	-1.19253	9.15E-08	8.97E-06	down
GNAZ	-1.1927	1.32E-05	0.000302	down
XLOC_008152	-1.19453	3.46E-07	2.18E-05	down
GPT2	-1.19577	5.60E-07	3.06E-05	down
PP12613	-1.19592	0.000172	0.0021	down
CECR1	-1.19596	2.25E-06	8.14E-05	down
LOC100131043	-1.19625	3.36E-05	0.000608	down
AZGP1	-1.19923	6.02E-05	0.000945	down
HPX-2	-1.19925	0.000271	0.002964	down
HAPLN3	-1.20137	9.26E-06	0.000229	down
XLOC_010319	-1.20218	0.019056	0.080153	down
ZNF132	-1.20258	2.20E-08	3.58E-06	down
SLC5A5	-1.20472	0.00085	0.007231	down
RIMS3	-1.20488	0.000158	0.001979	down
LOC100131015	-1.20637	1.95E-05	0.000403	down
CTIF	-1.20665	3.07E-06	0.000102	down
LOC100131034	-1.20853	3.09E-06	0.000103	down
FRMD1	-1.20859	1.66E-05	0.00036	down
DIRAS1	-1.21033	3.19E-05	0.000586	down
EMID2	-1.21298	1.16E-05	0.000274	down

MICALL1	-1.21523	7.92E-09	2.02E-06	down
NRIP3	-1.21617	0.000193	0.002288	down
GALT	-1.21715	6.83E-07	3.52E-05	down
LOC100506059	-1.21795	6.43E-05	0.000993	down
CERS4	-1.21833	5.38E-06	0.000155	down
GPRC5B	-1.21836	8.36E-06	0.000213	down
SLAMF7	-1.21853	3.59E-05	0.000634	down
ANKRD36B	-1.21955	0.000116	0.001573	down
PPP1R3C	-1.22197	5.67E-07	3.08E-05	down
PREX1	-1.22446	9.79E-13	7.85E-09	down
EYS	-1.2245	4.17E-06	0.000129	down
CNTD1	-1.22513	3.96E-06	0.000124	down
WISP2	-1.22514	0.000845	0.007201	down
MYEF2	-1.22646	1.50E-06	6.19E-05	down
PRSS33	-1.22887	0.000116	0.001573	down
SLC5A2	-1.23742	1.25E-06	5.40E-05	down
IL33	-1.23865	0.000127	0.001675	down
C5orf38	-1.23978	8.55E-05	0.001238	down
CYP4F22	-1.24117	0.001919	0.013665	down
LOC100507520	-1.24148	1.71E-05	0.000368	down
XLOC_002678	-1.2452	1.54E-07	1.28E-05	down
SLC9A7	-1.24598	4.07E-09	1.45E-06	down
LOC100192426	-1.24604	1.60E-06	6.46E-05	down
ZC3H12C	-1.24619	1.52E-07	1.28E-05	down
LOC100129840	-1.24883	0.000189	0.002256	down
CCDC149	-1.24939	2.83E-06	9.61E-05	down
ITGBL1	-1.25034	0.000238	0.002682	down
XLOC_011327	-1.25111	4.11E-05	0.000706	down
KIF1A	-1.25111	0.002498	0.016731	down
PPP4R4	-1.25149	7.61E-06	0.000198	down
LOC284578	-1.25159	1.76E-05	0.000375	down
PTGDR2	-1.25222	1.41E-05	0.000317	down
ZNF415	-1.25259	7.36E-06	0.000194	down
XLOC_006689	-1.25428	0.001518	0.011383	down
XLOC_001589	-1.25443	2.13E-06	7.81E-05	down
VEGFB	-1.25638	7.60E-07	3.76E-05	down
XLOC_002073	-1.25698	0.00065	0.005871	down
CD79A	-1.25791	1.06E-07	9.89E-06	down
CNIH3	-1.25898	0.000817	0.007014	down
C2orf74	-1.2597	1.18E-05	0.000277	down
IP6K3	-1.26062	3.05E-05	0.000567	down
BAIAP2	-1.26258	5.04E-05	0.000827	down
SLC9A7P1	-1.26431	3.45E-09	1.37E-06	down
XLOC_006035	-1.2649	2.08E-06	7.69E-05	down

HIST2H2BF	-1.26585	2.21E-06	8.04E-05	down
FBLN1	-1.26614	7.43E-05	0.001114	down
CUX2	-1.2665	2.84E-05	0.000537	down
SLC25A4	-1.27071	3.81E-07	2.33E-05	down
RYR2	-1.27469	3.10E-05	0.000573	down
SNTB1	-1.27528	4.09E-06	0.000128	down
APOH	-1.27572	0.006492	0.035098	down
INA	-1.27718	7.15E-07	3.62E-05	down
FER1L4	-1.27907	0.00042	0.004168	down
XLOC_l2_012919	-1.27945	6.58E-05	0.001012	down
FIGF	-1.27974	0.000109	0.001502	down
RAPGEF4	-1.28004	1.91E-05	0.000398	down
GC	-1.28057	0.001526	0.011424	down
LOC100505683	-1.28567	7.49E-05	0.001121	down
XLOC_l2_011744	-1.28573	3.22E-05	0.000588	down
RORA	-1.28822	2.05E-12	1.32E-08	down
ADRB2	-1.29121	0.000216	0.002485	down
GATA5	-1.29165	0.00038	0.003865	down
LYPD6B	-1.29218	8.77E-06	0.000221	down
ZSCAN22	-1.29581	2.35E-06	8.45E-05	down
XLOC_002487	-1.29813	6.08E-05	0.000952	down
CSF1R	-1.29933	1.29E-10	2.88E-07	down
KRT73	-1.3021	2.96E-07	1.96E-05	down
LOC730236	-1.30234	1.15E-07	1.05E-05	down
DUSP19	-1.30335	2.56E-05	0.000494	down
XLOC_007535	-1.30496	0.000242	0.002715	down
XLOC_008102	-1.30953	0.000461	0.004462	down
NLGN4X	-1.31149	5.14E-05	0.00084	down
RASGRP2	-1.31207	8.25E-07	4.00E-05	down
BASP1	-1.31254	1.65E-06	6.63E-05	down
HTR1E	-1.31298	1.38E-05	0.000311	down
PPARGC1A	-1.31398	7.52E-05	0.001124	down
CCDC110	-1.31786	3.16E-06	0.000105	down
DGKD	-1.31848	2.58E-09	1.18E-06	down
XLOC_012044	-1.31855	0.000179	0.002164	down
DCAF12L1	-1.32192	1.30E-05	0.000298	down
MAP7D2	-1.32315	9.65E-06	0.000237	down
MGC16025	-1.32383	0.000338	0.003528	down
CABP1	-1.32399	1.47E-05	0.000328	down
XLOC_002664	-1.32401	9.92E-05	0.001393	down
XLOC_007928	-1.32635	3.71E-05	0.000651	down
NFATC2	-1.32706	5.11E-07	2.91E-05	down
PPP3CC	-1.32727	7.29E-09	1.98E-06	down
TTN	-1.32782	3.44E-05	0.000618	down

TMEM132A	-1.32853	6.24E-05	0.000971	down
RAPH1	-1.32867	4.31E-06	0.000132	down
CXCR4	-1.32878	4.98E-06	0.000147	down
GSTT2B	-1.32935	1.75E-05	0.000372	down
IL11RA	-1.33026	8.18E-08	8.34E-06	down
PDE4A	-1.33191	2.99E-07	1.97E-05	down
CELA2B	-1.33269	0.001439	0.010907	down
CYP7B1	-1.33547	3.26E-06	0.000108	down
XLOC_l2_013972	-1.33668	0.000195	0.002301	down
CSRP2	-1.339	4.37E-06	0.000133	down
C8orf78	-1.34019	8.07E-07	3.93E-05	down
LOC728606	-1.34024	2.67E-05	0.00051	down
EPOR	-1.34334	6.38E-08	7.11E-06	down
NIM1	-1.3441	0.000114	0.001556	down
GKN2	-1.34602	0.00068	0.006087	down
ELL2	-1.34634	5.53E-08	6.62E-06	down
XLOC_006193	-1.34711	0.000315	0.003348	down
C11orf92	-1.35065	1.11E-06	4.93E-05	down
SARDH	-1.35128	3.60E-06	0.000116	down
IPO11	-1.35346	1.61E-06	6.48E-05	down
MAGEH1	-1.35374	6.15E-08	6.91E-06	down
CYB5R1	-1.35598	7.58E-09	1.99E-06	down
ATP11C	-1.3561	5.49E-07	3.03E-05	down
DKK1	-1.35781	0.000835	0.007135	down
APOC4	-1.35974	1.20E-06	5.22E-05	down
SMR3A	-1.35979	2.61E-05	0.000502	down
SNORD116-19	-1.36246	1.13E-06	5.01E-05	down
C10orf11	-1.36339	5.25E-07	2.95E-05	down
ALDH1L1	-1.3677	0.000188	0.002241	down
XLOC_008207	-1.36775	0.005637	0.03144	down
LOC100506235	-1.36787	2.29E-05	0.000455	down
LOC100144602	-1.36821	3.16E-05	0.000581	down
PTPRM	-1.36853	1.42E-08	2.83E-06	down
OSBPL6	-1.37089	6.96E-09	1.92E-06	down
GPR116	-1.37183	8.01E-05	0.001183	down
LOC389493	-1.37251	2.82E-05	0.000534	down
ARMCX1	-1.37381	1.48E-08	2.88E-06	down
SLC35F3	-1.37445	0.00041	0.004095	down
SYN2	-1.37539	3.65E-07	2.28E-05	down
XLOC_000757	-1.37763	0.000874	0.00737	down
MS4A1	-1.37867	0.000944	0.007864	down
RAB11FIP2	-1.37962	2.01E-07	1.54E-05	down
FERD3L	-1.38229	2.72E-06	9.32E-05	down
GFRA4	-1.38273	0.000166	0.002046	down

DUOXA1	-1.38288	4.72E-06	0.000141	down
CCL19	-1.38349	0.003172	0.020193	down
ASB11	-1.3845	3.00E-06	0.000101	down
C4orf39	-1.39161	0.001064	0.008614	down
LOC100287865	-1.39458	1.97E-05	0.000405	down
CCL16	-1.39492	2.86E-05	0.000539	down
PRKAA2	-1.39633	1.11E-05	0.000266	down
XLOC_010411	-1.39976	0.000208	0.002412	down
TMCO5A	-1.40158	4.99E-05	0.000821	down
CLDN19	-1.40365	1.97E-06	7.45E-05	down
SLC22A20	-1.40504	6.88E-06	0.000185	down
CELF4	-1.40735	3.29E-05	0.000599	down
IGFBP5	-1.41224	5.70E-05	0.000908	down
CYP2C8	-1.41337	0.000142	0.001832	down
LDHB	-1.41627	2.27E-06	8.19E-05	down
FAM110B	-1.41829	6.44E-07	3.40E-05	down
CXCR5	-1.42099	0.00201	0.014159	down
LINC00319	-1.42236	3.61E-06	0.000116	down
NR2F2	-1.42284	4.50E-08	5.80E-06	down
ADA	-1.42284	3.54E-05	0.000629	down
TP73-AS1	-1.42336	1.29E-07	1.13E-05	down
CTSF	-1.42396	1.07E-06	4.84E-05	down
CA9	-1.42464	2.57E-06	8.99E-05	down
LOC100507429	-1.42518	3.22E-05	0.000589	down
PLA1A	-1.42737	7.77E-07	3.83E-05	down
SCD5	-1.43022	7.09E-05	0.001073	down
LOC284244	-1.43206	2.05E-06	7.64E-05	down
COL6A5	-1.43213	2.46E-05	0.000478	down
XLOC_005458	-1.43261	7.44E-06	0.000196	down
ARHGEF17	-1.43294	7.00E-09	1.92E-06	down
ALDH3A1	-1.43625	3.60E-06	0.000115	down
MZB1	-1.43691	2.70E-05	0.000516	down
KRTAP5-7	-1.43805	0.000128	0.001689	down
COLEC12	-1.43806	1.88E-06	7.28E-05	down
XLOC_004600	-1.43874	0.000193	0.002288	down
XLOC_014403	-1.44165	2.00E-05	0.000409	down
GSTM2	-1.44217	2.39E-09	1.17E-06	down
LRRFIP1	-1.44238	7.21E-08	7.68E-06	down
LOC100289090	-1.44527	3.13E-05	0.000578	down
FZD4	-1.44641	5.83E-09	1.75E-06	down
LOC100507069	-1.44643	2.28E-05	0.000452	down
RGL3	-1.44683	5.47E-07	3.02E-05	down
XLOC_006283	-1.44729	2.49E-06	8.78E-05	down
XLOC_l2_004168	-1.44927	1.14E-06	5.01E-05	down

PLIN4	-1.45069	0.000699	0.006195	down
XLOC_002243	-1.45122	5.77E-05	0.000916	down
XLOC_007653	-1.45182	9.70E-05	0.001366	down
GPC3	-1.45195	0.000101	0.001415	down
NUPR1	-1.45325	6.93E-06	0.000185	down
SLC41A1	-1.45447	6.50E-08	7.19E-06	down
AMTN	-1.4546	0.028592	0.108141	down
UBE2QL1	-1.45612	2.41E-06	8.58E-05	down
LOC386758	-1.45864	2.70E-06	9.32E-05	down
GABARAPL1	-1.46048	2.81E-08	4.22E-06	down
ACSM2B	-1.46197	0.000125	0.001664	down
DPT	-1.46318	0.000115	0.001567	down
LOC100288273	-1.46345	0.003078	0.019718	down
DUX4L9	-1.4653	1.19E-06	5.22E-05	down
C3	-1.46602	0.000255	0.002833	down
LOC285095	-1.46742	1.01E-05	0.000245	down
C11orf85	-1.46982	0.000319	0.003383	down
TF	-1.47234	2.08E-05	0.000423	down
C13orf15	-1.47416	7.90E-08	8.22E-06	down
TAC4	-1.47485	4.34E-06	0.000133	down
BEX4	-1.47841	9.46E-09	2.25E-06	down
XLOC_000572	-1.48027	4.67E-06	0.00014	down
KLK11	-1.48146	0.000122	0.001637	down
SLC4A8	-1.4836	5.88E-05	0.000929	down
LDOC1	-1.48558	2.14E-07	1.61E-05	down
ITM2A	-1.4864	1.15E-07	1.05E-05	down
SLC13A3	-1.48944	1.93E-05	0.0004	down
LOC100132593	-1.48958	2.49E-05	0.000483	down
LOC388588	-1.4899	2.34E-06	8.42E-05	down
LOC100505853	-1.49067	5.81E-05	0.000921	down
ARHGAP24	-1.49235	5.53E-07	3.04E-05	down
XLOC_000555	-1.49256	6.19E-05	0.000965	down
CCKAR	-1.4955	1.85E-05	0.000387	down
RASSF2	-1.49878	5.63E-06	0.00016	down
SYNDIG1	-1.49907	0.000204	0.002377	down
NLRP14	-1.50106	2.14E-05	0.000431	down
XLOC_005851	-1.50231	2.15E-06	7.84E-05	down
XLOC_007832	-1.50419	3.52E-06	0.000114	down
RAP1GAP	-1.50454	6.80E-08	7.41E-06	down
XLOC_l2_008008	-1.50585	4.71E-05	0.000787	down
CHODL	-1.50647	4.40E-07	2.60E-05	down
FAM178B	-1.50652	1.21E-05	0.000282	down
OR12D2	-1.50678	3.50E-05	0.000625	down
XLOC_l2_011426	-1.50696	3.68E-05	0.000647	down

XLOC_001263	-1.5074	8.90E-06	0.000223	down
C16orf89	-1.50889	0.000615	0.00561	down
SCTR	-1.51712	1.28E-05	0.000295	down
COL4A4	-1.51733	7.39E-07	3.69E-05	down
SERPING1	-1.51786	4.20E-06	0.00013	down
APC2	-1.51848	3.96E-05	0.000686	down
XLOC_003156	-1.51865	4.32E-05	0.000732	down
PAIP2B	-1.5227	2.80E-08	4.22E-06	down
ERO1LB	-1.5255	0.000211	0.002443	down
PDGFD	-1.52803	1.87E-06	7.27E-05	down
GADD45B	-1.52991	3.97E-10	5.06E-07	down
MYBPC3	-1.53391	1.79E-07	1.43E-05	down
DUOX2	-1.5356	0.000213	0.002453	down
FCRL3	-1.54156	0.000474	0.004568	down
LOC645202	-1.54183	2.20E-05	0.000441	down
SCNN1B	-1.54559	0.00487	0.028062	down
XLOC_007604	-1.54658	2.13E-05	0.00043	down
EFHA2	-1.547	1.47E-05	0.000328	down
CECR7	-1.55056	1.74E-06	6.96E-05	down
AR	-1.55128	4.73E-07	2.75E-05	down
GGT7	-1.55189	2.12E-06	7.78E-05	down
LOC100506948	-1.55245	1.46E-08	2.87E-06	down
GSTT2	-1.55346	2.83E-06	9.61E-05	down
C22orf45	-1.55367	1.48E-05	0.000329	down
KRTAP2-1	-1.55813	6.04E-06	0.000168	down
C3orf18	-1.5623	4.32E-07	2.58E-05	down
LOC100507554	-1.56349	1.31E-05	0.0003	down
ADAMTS2	-1.56416	3.85E-06	0.000121	down
STK32B	-1.56826	4.88E-06	0.000145	down
MORN3	-1.56941	4.14E-06	0.000129	down
PRR21	-1.57017	5.81E-06	0.000163	down
PNOC	-1.57055	0.000396	0.003985	down
OR7A17	-1.57082	6.60E-06	0.000179	down
XLOC_004607	-1.57666	0.000175	0.002123	down
CELA3A	-1.57734	0.008968	0.045029	down
LINGO4	-1.57749	5.28E-07	2.96E-05	down
XLOC_013301	-1.5787	4.38E-05	0.00074	down
ZNF662	-1.57921	2.37E-05	0.000465	down
CDK14	-1.57975	7.63E-09	1.99E-06	down
XLOC_005514	-1.58055	4.98E-05	0.00082	down
GABRG2	-1.58055	8.15E-06	0.000209	down
SCNN1G	-1.58077	0.001444	0.01093	down
XLOC_001990	-1.58184	0.000937	0.007818	down
KCNE4	-1.58283	4.55E-05	0.000763	down

SOSTDC1	-1.58379	2.44E-05	0.000475	down
EVL	-1.58387	6.42E-13	6.86E-09	down
XLOC_l2_000356	-1.58449	2.22E-05	0.000443	down
PTPN13	-1.58587	8.13E-09	2.03E-06	down
PADI3	-1.59189	6.28E-06	0.000173	down
EYA2	-1.59212	1.94E-05	0.000402	down
LOC100131864	-1.59334	1.06E-05	0.000255	down
LOC100505754	-1.5936	2.90E-06	9.85E-05	down
C16orf47	-1.5942	8.19E-06	0.000209	down
LOC100507417	-1.59466	1.43E-05	0.000321	down
FUT1	-1.59521	1.19E-06	5.20E-05	down
KCNJ18	-1.5962	1.46E-05	0.000327	down
BEND5	-1.6001	2.90E-07	1.94E-05	down
RPP30	-1.60024	6.91E-07	3.55E-05	down
XLOC_001755	-1.60473	1.80E-05	0.00038	down
SOX21	-1.60696	0.00059	0.005424	down
ANKRD65	-1.60783	1.46E-07	1.24E-05	down
LMAN1L	-1.61189	1.33E-06	5.65E-05	down
IRF4	-1.61195	0.000255	0.002834	down
SYTL3	-1.61363	1.43E-11	5.75E-08	down
ANGPTL3	-1.61435	8.33E-07	4.02E-05	down
LOC255167	-1.6159	1.07E-06	4.84E-05	down
LOC393078	-1.61618	1.18E-05	0.000278	down
IGLL5	-1.61693	7.46E-06	0.000196	down
SERPINI1	-1.61789	2.57E-06	8.99E-05	down
SNORA5B	-1.61799	0.000177	0.002144	down
LOC100289134	-1.62482	2.60E-06	9.06E-05	down
MUC21	-1.62759	3.62E-06	0.000116	down
EPHB6	-1.62872	1.34E-05	0.000306	down
CHRNE	-1.63257	8.75E-07	4.17E-05	down
RIMS4	-1.63825	4.64E-06	0.000139	down
PCDH7	-1.64122	3.79E-05	0.000663	down
XLOC_000709	-1.6444	4.74E-05	0.000789	down
C8orf12	-1.64606	1.05E-05	0.000253	down
EIF5A2	-1.64743	3.07E-07	2.01E-05	down
KCNJ12	-1.64747	3.03E-05	0.000563	down
NPR3	-1.64929	1.75E-06	6.97E-05	down
LOC642648	-1.65077	2.76E-06	9.44E-05	down
XLOC_003719	-1.65178	3.44E-05	0.000618	down
CELA2A	-1.65625	0.000681	0.006091	down
XYLT2	-1.65675	4.18E-08	5.51E-06	down
TMEM211	-1.65719	6.09E-06	0.000169	down
PDIA2	-1.65795	0.000234	0.00265	down
NEAT1	-1.65884	6.84E-06	0.000184	down

TNFRSF17	-1.66028	0.000573	0.005297	down
GLUL	-1.66244	4.36E-08	5.66E-06	down
XLOC_010471	-1.66746	4.37E-05	0.00074	down
FMO2	-1.67256	1.64E-08	2.99E-06	down
CHRM3	-1.67272	6.92E-06	0.000185	down
NLRP7	-1.67852	0.00109	0.008774	down
GPR98	-1.68001	5.32E-07	2.98E-05	down
HOMER2	-1.68534	1.86E-07	1.46E-05	down
MUM1L1	-1.69323	1.85E-05	0.000387	down
GPR97	-1.69639	4.41E-07	2.60E-05	down
XLOC_001982	-1.69646	8.81E-07	4.19E-05	down
BEX1	-1.69887	0.000185	0.002221	down
CCNJL	-1.70189	5.54E-06	0.000158	down
MST1P9	-1.7023	2.10E-05	0.000426	down
P2RY4	-1.70374	9.58E-06	0.000236	down
LOC100506090	-1.70426	2.54E-06	8.92E-05	down
TCN1	-1.70459	0.000483	0.004639	down
XLOC_012007	-1.70585	3.82E-06	0.000121	down
LOC100506983	-1.70727	4.63E-07	2.71E-05	down
CHST11	-1.70732	4.18E-09	1.46E-06	down
EFEMP1	-1.70747	6.96E-09	1.92E-06	down
XLOC_l2_012552	-1.70838	6.90E-06	0.000185	down
SNRPN	-1.70947	9.27E-11	2.29E-07	down
RGN	-1.71336	1.21E-05	0.000282	down
C5	-1.71498	1.00E-08	2.29E-06	down
NAT8L	-1.71508	7.39E-07	3.69E-05	down
ENOX1	-1.71741	3.19E-07	2.06E-05	down
PHYHD1	-1.71746	2.26E-07	1.65E-05	down
XLOC_002647	-1.71836	9.06E-07	4.30E-05	down
XLOC_l2_006027	-1.71964	1.33E-05	0.000304	down
EGFEM1P	-1.72089	4.00E-06	0.000125	down
XLOC_001365	-1.72566	3.34E-05	0.000606	down
ASXL3	-1.72568	9.35E-07	4.37E-05	down
C10orf129	-1.73267	0.000104	0.001439	down
CYFIP2	-1.73295	6.15E-09	1.78E-06	down
CBS	-1.73804	7.87E-07	3.87E-05	down
LOC100128714	-1.73956	3.68E-06	0.000117	down
CXCL13	-1.74012	0.002366	0.016082	down
ZNF783	-1.74513	2.09E-05	0.000425	down
TPD52L1	-1.74572	3.86E-06	0.000122	down
TTC7B	-1.74667	6.21E-08	6.94E-06	down
XLOC_007374	-1.7499	1.52E-06	6.22E-05	down
LOC100505920	-1.75021	8.91E-06	0.000223	down
CARNS1	-1.75132	7.45E-05	0.001115	down

KANK4	-1.76046	1.09E-05	0.000261	down
XLOC_008013	-1.76084	6.98E-06	0.000186	down
REC8	-1.77077	3.84E-06	0.000121	down
LARP6	-1.77367	2.21E-07	1.62E-05	down
CHST3	-1.77713	1.71E-05	0.000368	down
LOC100653210	-1.77995	2.59E-05	0.0005	down
SNURF	-1.78036	1.91E-10	2.88E-07	down
PCDHA7	-1.7809	0.00011	0.001505	down
PPP2R3A	-1.78135	7.94E-08	8.22E-06	down
XLOC_011486	-1.78596	1.99E-05	0.000408	down
APLP1	-1.78907	2.74E-07	1.87E-05	down
XLOC_013195	-1.79	0.000102	0.001425	down
FIGN	-1.79694	8.56E-11	2.29E-07	down
OR2A25	-1.80324	6.63E-05	0.001019	down
CXCL17	-1.80673	1.89E-07	1.48E-05	down
FAM150B	-1.80968	6.86E-05	0.001045	down
XLOC_012786	-1.81285	4.06E-07	2.45E-05	down
HOXC4	-1.81308	1.55E-09	1.04E-06	down
HBZ	-1.81653	7.09E-06	0.000188	down
RNLS	-1.81805	8.33E-07	4.02E-05	down
PTPLA	-1.82197	1.42E-05	0.000318	down
XLOC_005201	-1.82833	4.90E-06	0.000146	down
SNAI3	-1.82963	2.01E-09	1.17E-06	down
BNIP3	-1.83049	4.76E-08	6.01E-06	down
KCTD8	-1.83084	3.66E-06	0.000117	down
XLOC_010356	-1.83222	4.22E-05	0.000719	down
LTF	-1.8328	0.00226	0.015522	down
BVES	-1.83686	5.03E-07	2.88E-05	down
LINC00317	-1.83722	5.26E-07	2.95E-05	down
C14orf37	-1.83914	1.23E-06	5.32E-05	down
RNF217	-1.83978	3.33E-08	4.75E-06	down
GRIA3	-1.84064	1.39E-05	0.000313	down
MAL	-1.84311	0.000442	0.004336	down
XLOC_l2_010759	-1.8449	2.80E-07	1.89E-05	down
XLOC_003695	-1.84677	9.65E-07	4.46E-05	down
SYNGR1	-1.85112	1.47E-09	1.02E-06	down
XLOC_001952	-1.85324	1.02E-06	4.66E-05	down
IRX3	-1.85413	2.17E-07	1.62E-05	down
OR10G4	-1.85924	1.19E-05	0.000278	down
KIAA0125	-1.85951	2.21E-05	0.000441	down
PAR-SN	-1.86381	1.46E-09	1.02E-06	down
DUOX1	-1.86814	7.72E-08	8.06E-06	down
XLOC_011849	-1.86887	1.16E-06	5.10E-05	down
ECHDC3	-1.8696	7.84E-09	2.02E-06	down

FAM124B	-1.87449	3.90E-06	0.000123	down
XLOC_012005	-1.8768	2.38E-07	1.71E-05	down
RXFP4	-1.87704	6.12E-05	0.000957	down
LOC100506965	-1.87781	1.51E-08	2.88E-06	down
XLOC_l2_003401	-1.88037	1.41E-06	5.90E-05	down
ZNF793	-1.8857	1.01E-08	2.29E-06	down
HYMAI	-1.88864	2.52E-07	1.77E-05	down
ODF3	-1.89242	6.41E-07	3.40E-05	down
LOC648691	-1.89384	4.22E-06	0.000131	down
XLOC_002094	-1.89688	5.88E-07	3.16E-05	down
RANBP3L	-1.89794	6.29E-06	0.000173	down
XLOC_011754	-1.89993	1.78E-06	7.02E-05	down
XLOC_013005	-1.90298	1.88E-06	7.28E-05	down
LEPR	-1.9121	1.04E-05	0.000251	down
XLOC_003123	-1.91211	2.72E-06	9.32E-05	down
XLOC_004446	-1.91228	2.05E-06	7.64E-05	down
C8orf71	-1.91518	5.15E-06	0.000151	down
CD3E	-1.9198	2.08E-09	1.17E-06	down
LOC100128893	-1.92469	2.38E-08	3.76E-06	down
FLJ42875	-1.92882	1.57E-08	2.93E-06	down
FAM165B	-1.93037	2.20E-07	1.62E-05	down
NPY	-1.93109	2.22E-07	1.62E-05	down
LOC100506342	-1.9325	1.88E-07	1.47E-05	down
SLC6A2	-1.93517	3.21E-06	0.000107	down
ADH7	-1.93669	0.000235	0.002658	down
XLOC_009343	-1.93834	4.43E-06	0.000135	down
CR2	-1.94153	0.000956	0.007935	down
LOC100507372	-1.95082	2.41E-08	3.79E-06	down
SLC2A4	-1.95711	8.90E-06	0.000223	down
AKNAD1	-1.96016	1.26E-06	5.40E-05	down
FAM167A	-1.96148	2.81E-06	9.56E-05	down
FCRL1	-1.97352	3.81E-05	0.000665	down
SPINK2	-1.97379	2.12E-05	0.000429	down
FSD2	-1.98141	8.43E-07	4.06E-05	down
ANKRD24	-1.98221	5.74E-06	0.000162	down
SMARCD3	-1.98478	9.75E-09	2.27E-06	down
AQP4	-1.9857	1.55E-07	1.29E-05	down
KCNIP3	-1.9865	2.99E-06	0.0001	down
ERP27	-1.98753	1.96E-05	0.000404	down
ACADL	-1.98997	1.73E-08	3.09E-06	down
CHAD	-1.99083	4.09E-06	0.000128	down
ADHFE1	-1.99249	2.92E-08	4.29E-06	down
XLOC_011535	-1.9969	6.60E-07	3.44E-05	down
XLOC_005371	-1.99708	2.99E-06	0.0001	down

LOC283856	-2.0013	1.55E-06	6.31E-05	down
WASF3	-2.00822	5.08E-08	6.31E-06	down
PLCL1	-2.00999	2.12E-09	1.17E-06	down
CD19	-2.01014	0.000116	0.001573	down
XLOC_003483	-2.01063	1.95E-06	7.40E-05	down
FAM151A	-2.01426	2.06E-06	7.65E-05	down
XLOC_013925	-2.01839	1.59E-07	1.31E-05	down
XLOC_003160	-2.03069	2.94E-06	9.92E-05	down
OR51I1	-2.03205	2.69E-06	9.31E-05	down
LOC654342	-2.03675	6.76E-07	3.50E-05	down
SPINK13	-2.0378	0.000268	0.002936	down
LOC400043	-2.03801	1.16E-09	8.87E-07	down
ST3GAL1	-2.03891	6.65E-10	7.09E-07	down
BTN1A1	-2.04041	4.82E-05	0.0008	down
CGNL1	-2.04451	6.16E-08	6.91E-06	down
GPER	-2.05026	5.23E-07	2.95E-05	down
GBGT1	-2.05242	1.78E-09	1.13E-06	down
XLOC_000247	-2.059	2.28E-06	8.23E-05	down
ERBB4	-2.06179	1.87E-06	7.27E-05	down
POPDC3	-2.08104	7.20E-08	7.68E-06	down
OR3A3	-2.08313	4.44E-06	0.000135	down
FBXL13	-2.08379	1.07E-06	4.82E-05	down
XLOC_012829	-2.10246	3.51E-08	4.94E-06	down
XLOC_010769	-2.10797	1.24E-06	5.37E-05	down
XLOC_003296	-2.11924	1.29E-05	0.000297	down
LIFR	-2.12481	2.95E-09	1.26E-06	down
XLOC_009144	-2.1324	1.93E-06	7.37E-05	down
RBPJL	-2.13688	1.42E-07	1.21E-05	down
XLOC_008945	-2.13795	2.17E-05	0.000436	down
XLOC_012662	-2.14028	8.26E-08	8.36E-06	down
PALM3	-2.14396	2.15E-08	3.55E-06	down
SLC1A2	-2.14623	1.44E-08	2.86E-06	down
FOLR1	-2.14887	5.40E-06	0.000156	down
PLCXD3	-2.16334	7.09E-07	3.60E-05	down
GALNT14	-2.17343	2.03E-06	7.59E-05	down
XLOC_l2_015752	-2.17437	2.05E-07	1.57E-05	down
TCEAL2	-2.17818	1.81E-07	1.44E-05	down
TMED6	-2.17913	3.93E-05	0.00068	down
XLOC_012670	-2.18168	4.00E-06	0.000125	down
XLOC_006529	-2.18198	5.96E-07	3.20E-05	down
BEX2	-2.18397	1.19E-08	2.50E-06	down
SCUBE2	-2.19185	2.54E-07	1.78E-05	down
LAMA2	-2.19303	9.56E-10	8.23E-07	down
ESRRG	-2.20072	1.71E-08	3.08E-06	down

NPHS1	-2.20098	3.69E-08	5.03E-06	down
PCDH9	-2.20681	1.29E-07	1.13E-05	down
MAST4	-2.21412	2.29E-06	8.26E-05	down
CACNA2D3	-2.22547	3.92E-09	1.43E-06	down
XLOC_010500	-2.22736	1.22E-08	2.53E-06	down
LYPD2	-2.23017	3.41E-06	0.000111	down
XLOC_012893	-2.23486	5.14E-06	0.00015	down
ORM1	-2.23579	5.08E-07	2.90E-05	down
PTPRZ1	-2.24153	4.23E-08	5.56E-06	down
LOC100128501	-2.24273	4.98E-07	2.86E-05	down
FAM20A	-2.24515	9.66E-09	2.26E-06	down
GAMT	-2.24613	3.84E-09	1.43E-06	down
RDH12	-2.24868	1.71E-06	6.86E-05	down
SLC16A7	-2.24935	3.97E-08	5.35E-06	down
GNMT	-2.25286	2.28E-08	3.64E-06	down
XLOC_001485	-2.2609	7.11E-06	0.000189	down
SERPINA4	-2.26143	5.99E-08	6.91E-06	down
LRRC17	-2.26188	4.26E-06	0.000131	down
PDE1B	-2.26275	3.75E-07	2.31E-05	down
KLHDC8A	-2.26419	7.53E-08	7.90E-06	down
ROBO3	-2.26651	2.22E-07	1.62E-05	down
ZMIZ1	-2.27217	1.32E-08	2.67E-06	down
LOC100507656	-2.27965	1.30E-06	5.56E-05	down
BLK	-2.28355	2.24E-09	1.17E-06	down
MRGPRX3	-2.30032	3.85E-08	5.24E-06	down
SLC26A7	-2.30701	1.21E-06	5.24E-05	down
LOC100240734	-2.30729	3.54E-09	1.37E-06	down
SLCO1A2	-2.30862	5.95E-06	0.000166	down
THSD4	-2.31563	4.41E-08	5.70E-06	down
LOC283731	-2.33037	7.85E-07	3.86E-05	down
RPRM	-2.33445	3.85E-07	2.34E-05	down
XLOC_005429	-2.33451	8.68E-08	8.65E-06	down
GUCA1C	-2.34054	1.20E-07	1.08E-05	down
XLOC_l2_010461	-2.34235	7.03E-07	3.59E-05	down
MYRIP	-2.34562	1.84E-09	1.13E-06	down
SLC2A12	-2.34855	1.83E-05	0.000385	down
PGCP1	-2.36135	2.79E-06	9.51E-05	down
GNRH2	-2.36239	1.83E-06	7.18E-05	down
LOC100506452	-2.36253	2.05E-09	1.17E-06	down
ZNF385B	-2.3652	2.05E-08	3.49E-06	down
WDR86	-2.38077	6.99E-07	3.57E-05	down
LOC100132790	-2.38607	2.73E-07	1.87E-05	down
DIRC3	-2.39805	3.34E-06	0.00011	down
TRIM50	-2.40157	1.96E-07	1.52E-05	down

XLOC_012240	-2.40245	1.98E-06	7.45E-05	down
ORM2	-2.4069	1.08E-07	9.99E-06	down
SERPINA5	-2.41195	9.90E-06	0.000242	down
CKB	-2.41446	2.16E-06	7.86E-05	down
SNORA80	-2.41531	2.41E-06	8.58E-05	down
CWH43	-2.41687	1.82E-05	0.000383	down
XLOC_004726	-2.44552	2.38E-07	1.71E-05	down
GPR133	-2.44557	2.52E-08	3.90E-06	down
PACSIN1	-2.44767	2.37E-07	1.71E-05	down
ENPP5	-2.45058	3.63E-08	5.03E-06	down
LINGO2	-2.45089	2.18E-08	3.56E-06	down
LOC400464	-2.47154	1.63E-07	1.33E-05	down
PSAPL1	-2.47295	2.77E-05	0.000527	down
OR52K2	-2.47433	3.42E-07	2.17E-05	down
EGFL6	-2.48808	3.67E-08	5.03E-06	down
GSTA3	-2.48931	3.19E-05	0.000585	down
PGC	-2.49946	1.66E-05	0.00036	down
XLOC_002302	-2.51048	6.81E-08	7.41E-06	down
C4orf7	-2.51202	0.001368	0.010493	down
EGFR	-2.51951	9.89E-07	4.53E-05	down
FGA	-2.52353	1.42E-06	5.91E-05	down
PP7080	-2.52571	3.66E-08	5.03E-06	down
XLOC_l2_001302	-2.53934	9.43E-07	4.40E-05	down
HSPB7	-2.54818	4.98E-07	2.86E-05	down
PTF1A	-2.55199	4.48E-10	5.13E-07	down
XLOC_l2_006789	-2.56501	2.82E-09	1.26E-06	down
XLOC_003780	-2.57289	1.84E-07	1.45E-05	down
KCNJ15	-2.57747	2.36E-09	1.17E-06	down
ODZ3	-2.58324	1.09E-07	1.00E-05	down
P2RX2	-2.58909	2.40E-07	1.71E-05	down
LOC100505601	-2.59242	8.17E-07	3.97E-05	down
LRCH3	-2.59251	1.32E-08	2.67E-06	down
C8orf84	-2.59577	9.75E-08	9.34E-06	down
PTGER3	-2.59711	1.49E-07	1.26E-05	down
FXYD4	-2.60608	2.64E-05	0.000507	down
NME9	-2.6069	1.07E-06	4.84E-05	down
XLOC_012210	-2.61401	1.26E-07	1.12E-05	down
HS3ST4	-2.61454	5.09E-09	1.60E-06	down
CDH2	-2.66674	1.54E-08	2.91E-06	down
RBPMS2	-2.66947	1.59E-08	2.93E-06	down
XLOC_005180	-2.67558	1.97E-06	7.45E-05	down
PAQR5	-2.67661	2.75E-08	4.19E-06	down
PDCD6	-2.7138	1.64E-07	1.33E-05	down
XLOC_006757	-2.71834	9.03E-08	8.96E-06	down

HPN	-2.74209	1.05E-07	9.78E-06	down
IGFALS	-2.74645	1.18E-08	2.48E-06	down
XLOC_005894	-2.75771	1.74E-07	1.39E-05	down
FABP3	-2.76737	7.38E-08	7.82E-06	down
XLOC_014216	-2.7719	2.16E-08	3.55E-06	down
XLOC_013835	-2.77232	1.49E-08	2.88E-06	down
EEF1A2	-2.77275	3.45E-07	2.18E-05	down
CSH1	-2.77481	2.28E-07	1.66E-05	down
LOC100505974	-2.83424	4.62E-09	1.56E-06	down
CLCNKA	-2.85139	1.72E-08	3.09E-06	down
G0S2	-2.85454	1.04E-07	9.78E-06	down
LOC150622	-2.85583	1.05E-08	2.32E-06	down
FGB	-2.87204	1.31E-07	1.14E-05	down
RTDR1	-2.87295	3.99E-08	5.35E-06	down
XLOC_002010	-2.90156	5.60E-08	6.67E-06	down
GREM2	-2.90289	2.08E-06	7.69E-05	down
LOC100128131	-2.90635	3.92E-08	5.31E-06	down
THOC6	-2.93886	7.07E-07	3.59E-05	down
XLOC_009894	-2.94124	2.55E-07	1.78E-05	down
C21orf30	-2.9423	6.03E-08	6.91E-06	down
FMO6P	-2.94409	5.36E-08	6.47E-06	down
ALB	-2.97474	4.62E-06	0.000139	down
XLOC_007161	-2.98599	2.20E-07	1.62E-05	down
KIF17	-3.00593	7.11E-08	7.65E-06	down
GRIA4	-3.00705	3.51E-08	4.94E-06	down
SUN5	-3.02187	1.31E-07	1.14E-05	down
HDC	-3.02246	5.87E-09	1.75E-06	down
IRX2	-3.03683	5.66E-06	0.00016	down
DNER	-3.0489	9.86E-08	9.36E-06	down
CLEC12B	-3.07531	7.97E-08	8.22E-06	down
SLC14A2	-3.08386	9.50E-07	4.42E-05	down
XLOC_009075	-3.0935	2.97E-09	1.26E-06	down
PLEKHM3	-3.14845	1.72E-07	1.38E-05	down
FNDC5	-3.1505	5.08E-08	6.31E-06	down
SLC26A9	-3.15381	4.59E-07	2.70E-05	down
MFSD4	-3.15587	1.00E-09	8.23E-07	down
XLOC_004339	-3.18061	1.04E-08	2.31E-06	down
PDILT	-3.21164	6.10E-08	6.91E-06	down
KCNK2	-3.2604	9.45E-07	4.40E-05	down
TMEM37	-3.26618	2.20E-09	1.17E-06	down
XLOC_001575	-3.29042	4.54E-08	5.82E-06	down
GHRL	-3.31449	4.38E-06	0.000134	down
CLIC6	-3.33013	9.35E-09	2.25E-06	down
SH3GL2	-3.33374	8.99E-09	2.19E-06	down

XLOC_009183	-3.34442	4.60E-08	5.88E-06	down
XLOC_l2_002611	-3.35175	1.13E-08	2.42E-06	down
XLOC_l2_013734	-3.35918	5.66E-08	6.72E-06	down
PLIN5	-3.39483	1.93E-08	3.30E-06	down
COL2A1	-3.4019	5.17E-07	2.94E-05	down
XLOC_006599	-3.44666	1.81E-08	3.13E-06	down
XLOC_014356	-3.45823	2.47E-07	1.74E-05	down
APOBEC2	-3.54878	2.03E-13	6.51E-09	down
KCNE2	-3.58361	6.71E-10	7.09E-07	down
XLOC_002258	-3.59105	5.71E-08	6.72E-06	down
SIGLEC11	-3.61712	8.48E-10	7.94E-07	down
PGA3	-3.66251	1.61E-10	2.88E-07	down
SULT2A1	-3.66383	1.77E-07	1.41E-05	down
CKMT2	-3.68406	5.94E-09	1.75E-06	down
UMODL1	-3.75145	2.62E-08	4.02E-06	down
B3GAT1	-3.80679	2.07E-08	3.50E-06	down
XLOC_004638	-3.81619	1.58E-08	2.93E-06	down
CCKBR	-3.85499	6.20E-09	1.78E-06	down
XLOC_002997	-3.91058	4.70E-09	1.56E-06	down
DRD5	-3.92081	4.80E-09	1.57E-06	down
LOC388796	-3.95479	2.98E-08	4.37E-06	down
GPR155	-3.9698	1.37E-10	2.88E-07	down
KCNJ16	-3.99564	1.91E-08	3.27E-06	down
XLOC_010491	-4.00742	1.12E-08	2.42E-06	down
PABPC1L2B	-4.05171	2.15E-08	3.55E-06	down
LIPF	-4.08609	6.41E-07	3.40E-05	down
GDF5	-4.11104	3.03E-09	1.26E-06	down
AGXT2L1	-4.24327	9.42E-08	9.15E-06	down
FGG	-4.27097	6.93E-08	7.48E-06	down
BCL2L11	-4.3045	2.57E-08	3.97E-06	down
SSC5D	-4.37581	5.31E-08	6.43E-06	down
XLOC_l2_000080	-4.52382	4.85E-09	1.57E-06	down
CPA2	-4.66347	2.37E-09	1.17E-06	down
CKM	-4.74148	4.33E-08	5.66E-06	down
LOC100507464	-5.02664	6.54E-08	7.21E-06	down
LOC100506412	-5.12266	3.85E-10	5.06E-07	down
SNORA5A	-5.2152	1.23E-07	1.10E-05	down
TMEM184A	-5.33138	1.80E-08	3.13E-06	down
XLOC_010144	-5.39546	2.52E-08	3.90E-06	down
CHIA	-6.37686	3.12E-08	4.54E-06	down
GIF	-7.08042	1.34E-11	5.75E-08	down
ATP4A	-7.14004	2.06E-10	2.88E-07	down
ATP4B	-7.23622	9.28E-11	2.29E-07	down
